# CDC42-effector interaction inhibitors alter patterns of vessel arborization in skin and tumors *in vivo*

**DOI:** 10.1016/j.isci.2025.112971

**Published:** 2025-07-14

**Authors:** Linh M. Vuong, Stephanie Hachey, Jessica Shiu, Danny F. Xie, Noel Salvador, Nicoletta Brindani, Sine Mandrup Bertozzi, Maria Summa, Rosalia Bertorelli, Andrea Armirotti, Rachel Pham, Vance S.H. Ku, Swara D. Limbekar, Terry Nguyen, Bernard Choi, Christopher C.W. Hughes, Marco De Vivo, Anand K. Ganesan

**Affiliations:** 1 Department of Dermatology, University of California, Irvine, Irvine 92697, CA, USA; 2 Department of Molecular Biology and Biochemistry, University of California, Irvine, Irvine 92697, CA, USA; 3 Beckman Laser Institute and Medical Clinic, University of California, Irvine, Irvine 92697, CA, USA; 4 Department of Biological Chemistry, University of California, Irvine, Irvine 92697, CA, USA; 5 Molecular Modeling and Drug Discovery Lab, Istituto Italiano di Tecnologia, 16163 Genova, Italy; 6 Analytical Chemistry Facility, Istituto Italiano di Tecnologia, 16163 Genova, Italy; 7 Translational Pharmacology Facility, Istituto Italiano di Tecnologia, 16163 Genova, Italy

**Keywords:** microenvironment, pharmacology, cancer;

## Abstract

Skin tumors require a vascular supply to grow beyond 1 mm in depth, yet existing anti-angiogenesis agents are largely ineffective at treating melanoma tumors arising in skin. Using an approach that integrates antibody infusion, optical tissue clearing, multiphoton imaging, and vessel tracing, we identified the CDC42 GTPase RhoJ as a critical regulator of skin vessel arborization. Small molecules that target both RhoJ and CDC42 (CDC42 interaction inhibitors), but not those that target only CDC42 (CASIN), inhibit vessel branching in mouse skin *in vivo* and vascular organoids *in vitro*. This anti-vascular effect was not limited to skin, as CDC42 interaction inhibitors blocked melanoma tumor vascularization and inhibited tumor growth to a similar degree as Braf inhibitors. Taken together, this work identifies small molecules that target RhoJ as selective tumor anti-vascular agents. RhoJ-targeting drugs have a particular proclivity for blocking skin vascularization, nominating them as new treatments for inflammatory/vascular skin disease.

## Highlights

• Optical clearing and 3D tracing can quantify vessel arborization in skin and tumors
• RhoJ plays a selective role in controlling vessel arborization in skin *in vivo*
• CDC42 interaction inhibitors block vessel arborization within melanoma tumors
• CDC42 inhibitors that target RhoJ can inhibit skin vessel arborization *in vivo*

## Graphical abstract

**Figure:**
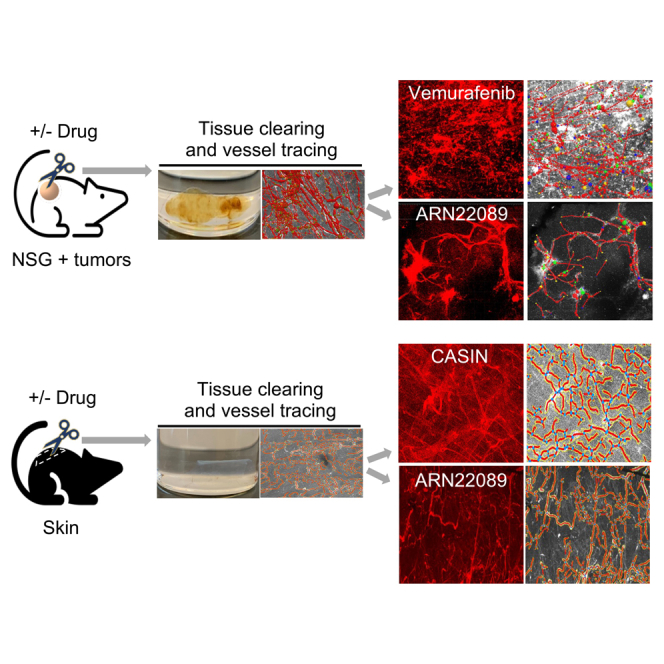

## Introduction

Vascular development and maintenance is a process requiring dynamic rearrangement of the endothelial cell cytoskeleton^1^^,^^2^^,^^3^ in order to properly respond to environmental cues.^4^ This process is critical not only for vascular homeostasis in the skin and other tissues^5^ but also for tumors, particularly those that arise in the epidermis where there is a paucity of existing vasculature.^6^^,^^7^^,^^8^ Tumors accelerate angiogenesis over what is observed in normal tissues by producing a cornucopia of angiogenesis-promoting factors, resulting in the formation of a haphazard, tortuous vessel tree that is unable to deliver nutrients evenly to the tumor.^9^^,^^10^

Recent technologic advances have developed methods combining optical clearing with semi-automated tracing to visualize blood vessels in tissue.^11^^,^^12^^,^^13^ This approach has been recently applied to tumors,^14^^,^^15^ revealing a tumor vasculature that is characterized by large tortuous vessels, leaky basement membranes, and unperfused voids.^16^^,^^17^ Other studies utilized longitudinal imaging approaches to provide structural insight into how vessels develop or repair after injury.^5^^,^^18^ These approaches have not yet been applied to study skin vasculogenesis, inherited skin vascular anomalies,^19^^,^^20^ or melanoma.^6^ It is critically important to understand vasculogenesis in the skin, as existing anti-angiogenic therapies are many times toxic or only minimally effective in skin tumors^21^ and have not yet been broadly tested in skin disease.^22^

Part of the challenge in identifying potent anti-vascular agents for skin disease or skin cancer lies in the fact that many intracellular signaling pathways can activate angiogenesis.^23^ Vascular endothelial growth factors (VEGFs), which activate intracellular signaling cascades that induce actin remodeling and focal adhesion assembly,^24^ are major chemo-attractants for migrating endothelial cells, and spatial activation of VEGFR2 signaling is critical in the maintenance of the adult vasculature.^5^ Semaphorins have also been identified as regulators of vasculogenesis—Semaphorin 3E binds to the PlexinD1 receptor to induce receptor internalization, focal adhesion disassembly, and, subsequently, vessel regression.^25^^,^^26^ CDC42 GTPases, RhoJ and CDC42, are critical mediators of both attractive and repulsive cues generated by activation of VEGF and Plexin receptors, respectively.^27^ RhoJ, a protein with 55% homology to CDC42, is highly expressed in endothelial cells and induces actin depolymerization, focal adhesion disassembly, and endothelial cell contraction.^28^^,^^29^ CDC42, in contrast, activates focal adhesion assembly and stimulates the migration of endothelial cells.^30^^,^^31^^,^^32^^,^^33^ RhoJ deficiency results in impaired VEGF-induced endothelial cell migration,^34^ impaired retinal vascular angiogenesis early during development,^35^^,^^36^ and impaired tumor angiogenesis.^37^ However, it is currently unclear what distinct roles RhoJ/CDC42 play in vasculogenesis in skin or tumors.

Targeting RhoJ/CDC42 with small molecules has been difficult secondary to their globular structure and lack of readily apparent druggable pockets.^38^^,^^39^ However, two approaches to target this family of GTPases have recently been developed. The first strategy involves blocking the ability of these GTPases to interact with guanine nucleotide exchange factors.^40^ The Cdc42 Activity-Specific INhibitor (CASIN) is a small molecule that specifically blocks CDC42 GTP exchange without affecting GTP exchange of other CDC42 family members such as RhoJ.^40^ *In vivo* administration of CASIN promotes hematopoietic stem cell mobilization,^41^ induces skin thickening,^42^ extends the lifespan of experimental animals,^43^ and modulates T regulatory cell function in tumors.^44^ We recently used a structure-based drug design approach to discover a new class of small molecules that block interactions between RhoJ and CDC42, and their downstream effectors as the effector interaction interface is conserved between these GTPases.^45^^,^^46^ These agents blocked S6 and extracellular signal-regulated kinase (ERK) activation, inhibited angiogenesis in vascularized tumor organoids, and inhibited the growth of mouse and patient-derived tumors *in vivo.*^45^^,^^46^ Notably, this *in vivo* activity was distinct from that reported for CASIN, which is not known to affect angiogenesis, suggesting that RhoJ and CDC42 may play distinct roles in these processes.

Here, we sought to determine how RhoJ and drugs that inhibit CDC42-effector interactions modulate angiogenesis in skin and tumors *in vivo*. We developed a platform combining tissue clearing and vascular labeling with fluorescent antibodies and 3D semi-automated vessel tracing to discover that RhoJ-deficient mice had reduced vessel arborization in their skin with no other signs of overt toxicity. We applied this new imaging approach to patient-derived melanoma xenografts treated with CDC42-effector interaction inhibitors or other standard-of-care melanoma therapies (BRAF inhibitors) and demonstrated that both types of drugs had similar tumor-suppressive effects and altered patterns of vessel arborization in tumors. We also find that CDC42 interaction inhibitors can alter vessel arborization patterns in the skin of wild-type (WT) but not RhoJ-knockout (KO) mice. Notably, the effects of these agents are greater than that observed for VEGF inhibitors, and the selective CDC42 inhibitor CASIN does not alter skin vessel arborization patterns. The effect of CDC42 interaction inhibitors on human blood vessels was confirmed *in vitro* using humanized organotypic micro-physiological systems (vascularized micro-organ [VMO] and vascularized microtumor [VMT]) that recapitulate *in vivo* neo-vascularization in normal tissues and tumors, respectively. Taken together, these studies identify a group of selective anti-angiogenesis agents that target RhoJ signaling.

## Results

### RhoJ deletion disrupts the vascular network in the skin of RhoJ-KO mice

**Figure 1:**
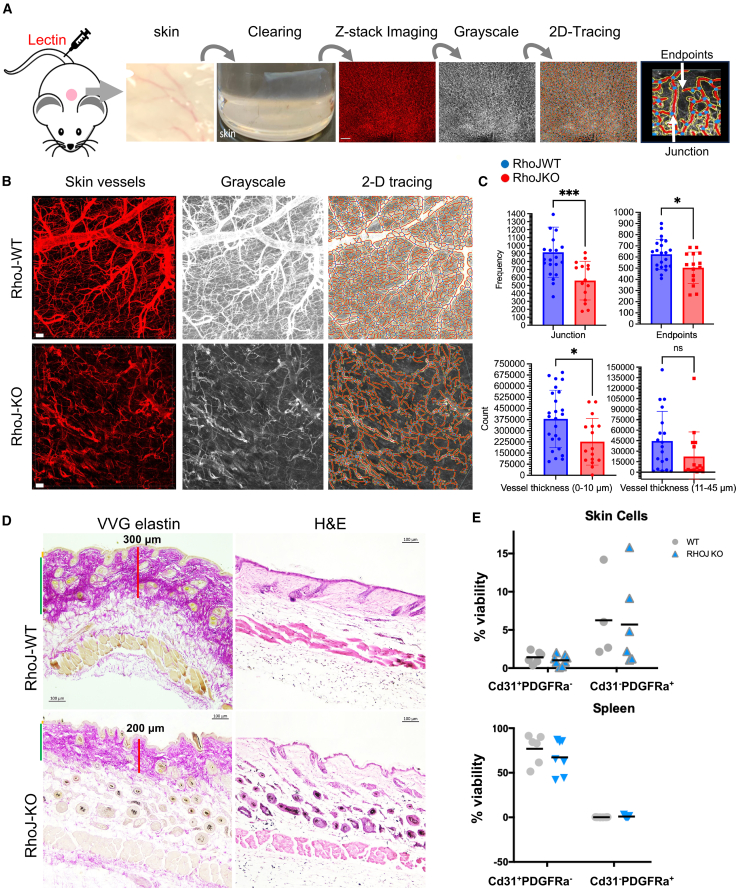
RhoJ regulates skin vessel arborization in skin (A) Diagram of optical tissue clearing and vessel analysis. Each image (1.107 × 1.107 mm; 1,024 × 1,024 pixels) has >50 z-stacks (5 μm/stack). z-stacks are compressed into 2D Tiff file, converted to grayscale, and analyzed with AngioTool. Images were generated from three mice per group. (B) Representative images of skin vasculature from RhoJ wild-type (WT) and knockout (KO) mice. Fluorescence images of lectin-labeled structures were obtained, converted to grayscale, and traced with AngioTool. Scale bar is 50 μm. (C) Scatter bar plot of the frequency of vessel branches, termini, and vessel diameters observed in (B); ^∗,∗∗∗^*p* < 0.05, 0.0005; dots on each plot correspond to the number of image stacks analyzed with at least four images analyzed per group, *n* = 3 mice per genotype. Data representative as mean ± SD. (D) VVG elastin and H&E staining of skin from RhoJ-WT and RhoJ-KO mice. Note the presence of an intact epidermis and hair follicles indicating that RhoJ deletion did not induce skin necrosis. (E) Scatterplot showing the percentage of Cd31^+^ or Pdgfra^+^ live cells in the skin and spleen from ≥4 mice per group (RhoJ-WT and RhoJ-KO). Data representative as mean ± SD.

RhoJ-KO mice have decreased branching of the retinal vasculature during development, a phenotype that improves with age.^27^ RhoJ is also known to play a role in adult vascular homeostasis, as RhoJ-KO mice had impaired tumor angiogenesis^37^ and impaired skin wound healing.^47^ However, the effects of RhoJ deletion on vascular homeostasis in adult skin has never been measured. Initial work sought to examine if RhoJ-KO mice had altered vascular architecture in the skin as compared to RhoJ-WT mice. We previously combined tissue clearing with intravascular infusion of DyLight 649-labeled tomato lectin to visualize normal vasculature in the brain.^48^^,^^49^ Here, we modified an existing immunolabeling three-dimensional imaging of solvent-cleared organs^50^ protocol to clear skin tissue harvested from RhoJ-KO and WT mice infused with tomato lectin prior to sacrifice (Figure 1A). Cleared skin was imaged with a Leica SP8 multiphoton microscope to generate z stack images of skin and measure differences in vessel architecture between WT and RhoJ-KO mice using two different analytic pipelines. A gross comparison of flattened images generated from RhoJ-KO and WT mouse skin revealed that RhoJ-KO mice had a decrease in the number of skin vessels (Figure 1B). Differences in vascular architecture in the skin were quantified by two different methods. AngioTool is a software package that takes 2D images, quantifies pixels in grayscale, and measures junctions and endpoints. This package has been used to measure differences in the vascular architecture of the murine embryonic hindbrain, post-natal retina, and allantois explant.^51^ A second pipeline used here, neuTube, processes mulitple images of a sample each focused at a slightly different depth (i.e. a z-stack) to generate a 3D image. The program uses these z-stacks to trace neurons and can quantify terminal nodes, branch nodes, and the total number of neurons.^52^ This pipeline has only recently been used to trace vessels. We compared the ability of these two methods to detect differences in vessel architecture in RhoJ-KO and RhoJ-WT skin. AngioTool analysis revealed that the skin of RhoJ-KO mice had fewer vessel junctions and endpoints as compared to RhoJ-WT skin (Figure 1C, top), and these phenotypes were reproducible across animals (Figure S1D). Analysis of the same images with neuTube revealed that RhoJ-KO skin had fewer terminal nodes, branch nodes, vessel branches, and number of vessel segments as compared to RhoJ-WT skin (Figures S1A–S1C). We also compared the distribution of vessel sizes in RhoJ-WT and RhoJ-KO skin. RhoJ-KO skin had significantly fewer capillaries (0–10 μm diameter) and less arterioles/venules (between 11 and 45 μm diameter), although this change was not statistically significant (Figure 1C, bottom). Despite the observed decrease in vessel density, RhoJ-KO skin showed no obvious pathological changes in the epidermis and dermis as visualized by hematoxylin and eosin staining (H&E) (Figure 1D, right). We did, however, observe a slight decrease in thickness of the superficial dermal layer of RhoJ-KO skin, as highlighted by a Verhoeff-Van Gieson (VVG) stain (Figure 1D, left). Flow cytometry analysis revealed that RhoJ-KO skin and RhoJ-WT skin had a similar number of endothelial cells (CD31^+^ cells^53^) and fibroblasts (PDGRα^+^ cells^54^) in their skin (Figure 1E, top) and spleen (Figure 1E, bottom). Taken together, these results indicate that while RhoJ deletion affects vascular arborization in the skin, it does not grossly affect skin structure or the viability of fibroblasts or endothelial cells. Of note, we show here that 3D quantitative approaches can capture differences in the arborization of the vasculature in mice to a similar extent as 2D methods, motivating us to apply this system to measure vascular changes in cleared tumors, which are thicker structures. In sum, these studies suggest that inhibiting RhoJ could alter skin vessel arborization patterns.

### CDC42 inhibitors alter vascular arborization patterns in patient-derived xenografts implanted into mice

**Figure 2:**
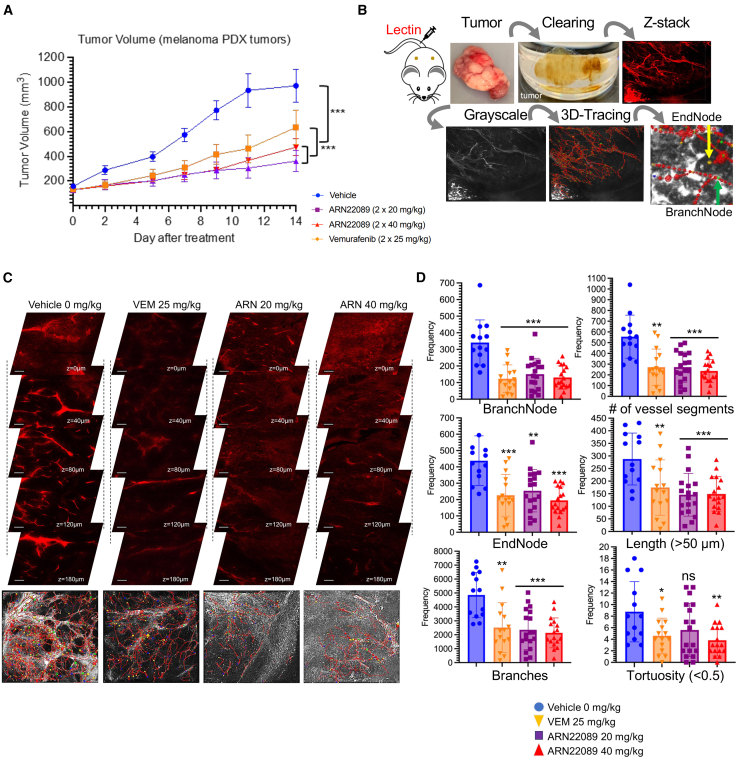
CDC42 inhibitors alter vascular arborization patterns in tumors (A) Plot of tumor volume as a function of time in mice treated with vehicle (blue line), vemurafenib (orange line), or ARN22089 (purple [20 mg/kg] and red [40 mg/kg] lines) at the indicated doses. Graph represents mean ± SEM. ^∗∗∗^*p* value < 0.0005 (vehicle vs. inhibitors; 25 mg/kg vemurafenib vs. 20 and 40 mg/kg ARN22089), *n* = 6 mice per group. (B) Schematic diagram of tissue clearing, visualization, and vessel analysis of the tumors. Each image (1.107 × 1.107 mm; 1,024 × 1,024 pixels) has >200 z-stacks (5 μm/stack). (C) Representative z-stacks of fluorescent images from tumors in mice treated with vehicle, vemurafenib (25 mg/kg twice daily), or ARN22089 (20 and 40 mg/kg twice daily) orally are shown. Bottom represents corresponding grayscale vessel tracing images; scale is 100 μm. (D) Scatterplot with bar graphs show the statistical quantification of vessel parameters (number of branchpoints/endpoints and branching, number of vessels, length, and tortuosity) after 3D vessel tracing for >3 tumors per group. Data representative as mean ± SD. Dots correspond to the number of image stacks that were analyzed, with ≥4 images analyzed per tumor. ^∗,∗∗,∗∗∗^*p* < 0.05, 0.01, 0.0005. Effects were compared between vehicle- and drug-treated mice.

Previous work discovered a new class of CDC42 interaction inhibitors that could inhibit tumor growth *in vivo*^45^^,^^46^ and block angiogenesis in VMTs *in vitro.*^46^ Initial work sought to examine whether the lead compound in our newly discovered class of CDC42 inhibitors (ARN22089)^46^ could inhibit the growth and vascularization of melanoma patient-derived xenografts. A melanoma patient-derived xenograft was implanted into the flanks of NSG mice, and when the tumors reached a volume of 150–200 mm^3^, they were treated with the indicated doses of the CDC42 inhibitor ARN22089, vemurafenib, or vehicle twice daily for 2 weeks by oral gavage (Figure 2A). We observed that ARN22089 inhibited the growth of tumors as effectively as vemurafenib. To compare how ARN22089 and vemurafenib affected arborization of vessels in tumors, we sacrificed the mice after 2 weeks of treatment and used our vascular imaging approach to quantify vascular branching in 3D using the neuTube pipeline (Figure 2B). Gross examination of stacked images from drug-treated mice revealed a decrease in vasculature in treated mice as compared to controls (Figure 2C, top). NeuTube-based quantification of vessel architecture (Figure 2C, below) revealed that ARN22089 affected vessel number and branching to a similar degree as vemurafenib. NeuTube analysis of images revealed that ARN22089 had a dose-responsive effect on the number of branches, the number of terminal nodes, vessel tortuosity, and vessel length (Figure 2D), and these results were reproducible across animals (Figure S2A). ARN22089 and vemurafenib had similar effects on vessels of all sizes (≤20, between 20 and 50, and >50 μm), with the greatest impact on the smaller vessels (Figures S2B and S2C). It is important to note that the vessels observed in tumors were in general larger and more tortuous than those seen in skin, consistent with studies published by others.^16^^,^^17^ To verify that the imaging results correlated with standard histologic methods, we stained ARN22089-treated tumors with H&E and CD31, revealing that ARN22089-treated tumors had fewer CD31-stained vessels (Figure S2D). We also performed daily tail vein injection of a second CDC42 lead inhibitor (ARN25062) and ARN22089 and observed that both compounds stunted tumor growth (Figure S3A) and reduced vessel arborization in tumors grossly (Figure S3B). Additionally, vessel arborization changes induced by both analogs could be detected using both AngioTool and neuTube (Figures S3C and S3D).

### CDC42 interaction inhibitors block angiogenesis in human-derived VMTs *in vitro*

**Figure 3:**
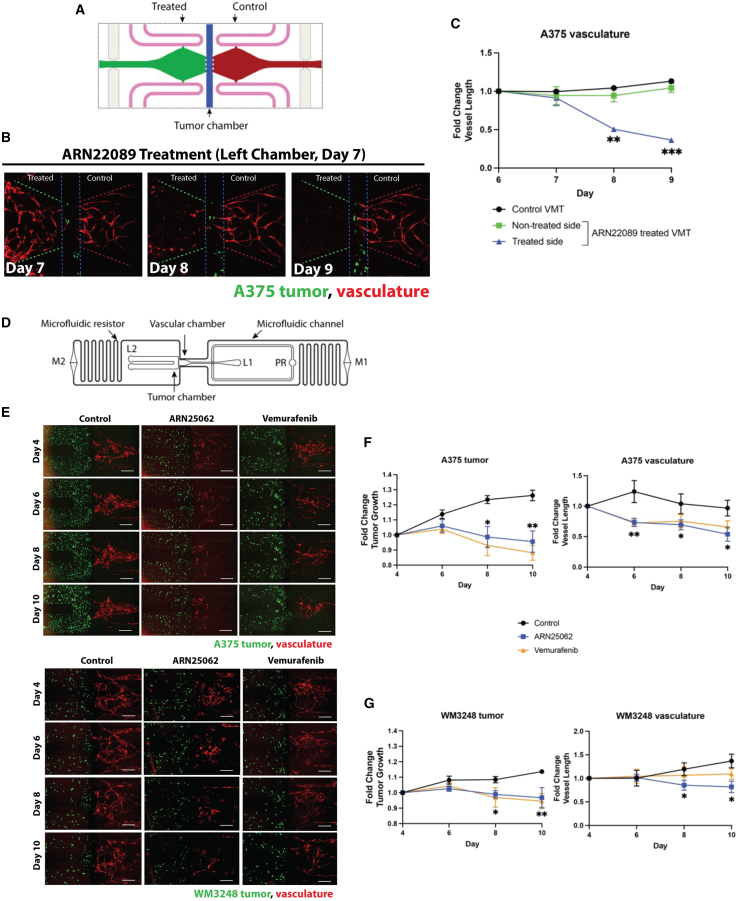
CDC42 inhibitors disrupt vasculature in tumor organoids (A) Diagram showing a double-chamber microfluidic design where two independently fed vascular chambers (green and red) flank a central 200-μm-wide tumor chamber (blue) separated by 3 PDMS posts on each side. The left vascular chamber (green) was treated with ARN22089 (2 μM) on day 6, while the right side received vehicle only (control, red). (B) Representative fluorescent micrographs of double-chamber VMT with A375 (green) in the center and vasculature (red) on either side. (C) Quantification of A375 tumor-associated vasculature showing fold change in vessel length from baseline for left side (treated) vs. right side (control). Data representative as mean ± SEM. (D) Schematic showing a single dual-chamber microfluidic device. The vascular chamber connects to the tumor chamber (800 μm wide), separated by 6 PDMS posts spaced 50 μm apart that serve as burst valves to prevent the gel from traversing the chamber. EC and lung fibroblast (LF) are introduced into loading port L1, and cancer cells are introduced separately into loading port L2. Loading is facilitated by a pressure regulator (PR). Tissues are maintained via hydrostatic pressure generated across microfluidic channels connecting media reservoirs M1-M2. Physiological flow rates are established by microfluidic resistors. (E) Representative fluorescent micrographs of dual-chamber VMT containing A375 or WM3248 tumor (green) and vasculature (red), treated with control (vehicle only), ARN25062 (2 μM), or vemurafenib (2 μM) for 48 h starting on day 4. Media were refreshed on day 6, day 8, and day 10. Scale bars, 500 μm. (F) Quantification of A375 tumor growth and tumor-associated vasculature in the VMT showing fold change from baseline ^∗^*p* < 0.05, ^∗∗^*p* < 0.01. (G) Quantification of WM3248 tumor growth and tumor-associated vasculature showing fold change in tumor vessel length from baseline. ^∗^*p* < 0.05, ^∗∗^*p* < 0.01. Data representative as mean ± SEM.

Previous work had demonstrated that ARN22089 could inhibit vessel growth in human-derived VMTs *in vitro* using single-chamber VMT models.^55^ Tumor cells in these models maintain (1) their *in vivo* gene expression profile, (2) physiologic cell-cell interactions, and (3) responsiveness to anti-cancer drugs.^56^^,^^57^ To determine whether CDC42 interaction inhibitors had direct effects on tumor vasculature, we used a second VMT platform design with independently treated vascular chambers and an intervening tumor chamber (Figure 3A) so that one could measure the effects of drugs after infusion on one side of the chamber versus the infusion of the other with vehicle. ARN22089 disrupted the vasculature in drug-treated but not vehicle-treated chambers with a concomitant effect on tumor growth (Figure 3B), similar to the effects that were observed previously.^46^ We noted that ARN22089-treated chambers had shorter vessels (Figure 3C) as measured by REAVER (MATLAB).^58^ Next, we sought to examine whether ARN25062 and vemurafenib could also inhibit vessel growth in a dual-chamber microfluidic device that consists of a vascular network adjacent to a second chamber containing the tumor, modeling ingrowth/cooption of vessels by tumors (Figure 3D). In the dual-chamber VMT, A375 tumors and associated vascular structures regressed significantly in response to treatment with ARN25062 and vemurafenib (Figure 3E, top), as indicated by a reduction in tumor growth on days 8 and 10 and decreased vessel length at each time point (Figure 3F). Notably, there was no significant difference in response between ARN25062 and vemurafenib in the VMT. Similar results were observed for the melanoma cell line WM3248; we observed a significant reduction in tumor growth and vessel length with ARN25062 and vemurafenib treatment, while a significant decrease in vessel length was only observed in ARN25062-treated organoids (Figure 3E, bottom and 3G). These results indicate that CDC42 inhibitors block vessel elongation *in vitro* and prompted us to further examine whether such vascular-specific effects of the drug could also be observed in the absence of tumors.

### CDC42 interaction inhibitors disrupt skin vasculature in mice *in vivo* and in human-derived VMOs *in vitro*

**Figure 4:**
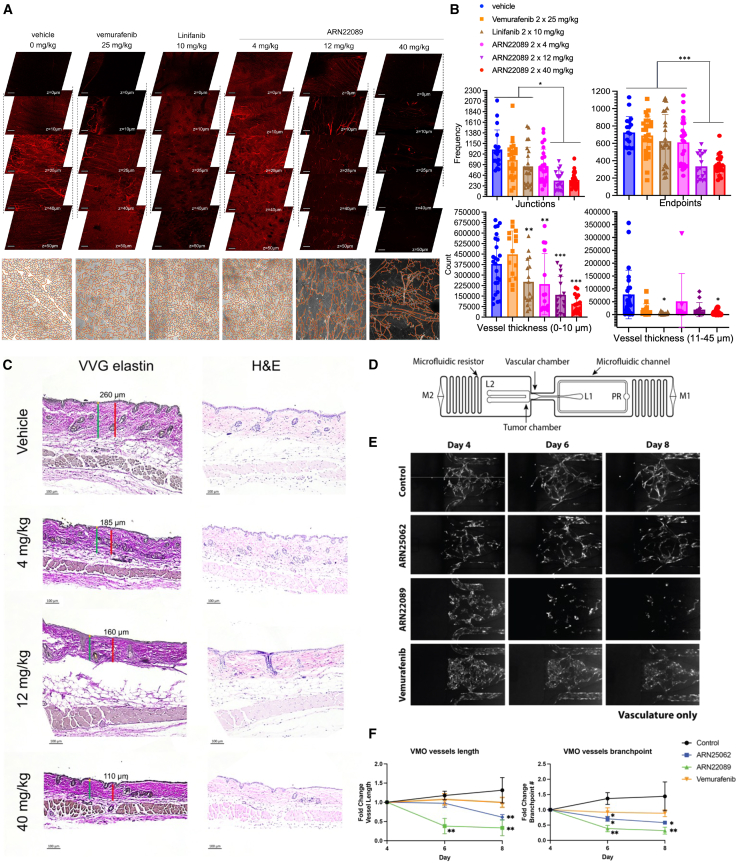
CDC42 inhibitors alter vessel arborization in skin (A) Skin of mice treated with vehicle, vemurafenib, linifanib, and ARN22089 at the indicated doses were cleared and imaged to generate z-stacks as described, with representative stack shown. Bottom of each stack includes the 2D AngioTool tracing of pixels in grayscale. Scale bar is 100 μm. Each image (1.107 × 1.107 mm; 1,024 × 1,024 pixels) has >50 z-stacks (5 μm/stack). (B) Scatterplot with bar graphs shows the frequency of branches, termini, and number of vessels with different thickness quantified from flattened images using the 2D AngioTool and Fiji software. Two-way ANOVA comparison, >3 mice per condition, and dots on graphs correspond to each image stack. ^∗,∗∗,∗∗∗^*p* < 0.05, 0.01, 0.0005; comparison to vehicle or indicated by the bar lines. At least four images analyzed per condition. Data representative as mean ± SD. (C) VVG elastin and H&E staining of skin from WT and RhoJ-KO mice. Similar to the RhoJ-KO, the presence of an intact epidermis and hair follicles are observed in all samples, with a slight decrease in VVG highlighted areas in treated animals. (D) Schematic showing a single dual-chamber microfluidic device. The vascular chamber is 800 μm wide, separated by 6 PDMS posts spaced 50 μm apart that serve as burst valves to prevent the gel from traversing the chamber. EC and LF are introduced into loading port L1. Loading is facilitated by a pressure regulator (PR). Tissues are maintained via hydrostatic pressure generated across microfluidic channels connecting media reservoirs M1-M2. Physiological flow rates are established by microfluidic resistors. (E) Representative fluorescent micrographs of a dual-chamber VMO showing vasculature (greyscale), treated with control (vehicle only), ARN25062 (2 μM), ARN22089 (2 μM), or vemurafenib (2 μM) for 48 h starting on day 4. Media were refreshed on day 6, day 8, and day 10. (F) Quantification of VMO-associated vasculature showing fold change in vessel length and number of branchpoints compared to baseline. ^∗^*p* < 0.05, ^∗∗^*p* < 0.01. Data representative as mean ± SEM.

RhoJ-KO mice had notable changes in the vascular arborization of skin compared to WT mice (Figure 1), suggesting that inhibiting RhoJ signaling could affect skin vasculogenesis. We next compared the effects of ARN22089, vemurafenib, or linifanib treatment on the arborization of vessels in the skin of WT mice (Figure 4). For these experiments, C57B6 mice were treated with ARN22089 (4, 12, or 40 mg/kg), vehicle, 25 mg/kg vemurafenib, or linifanib (10 mg/kg) twice daily for 1 week, skin was harvested and prepped for our imaging approach, and vessel architectural changes were quantified using AngioTool. Gross examination of image stacks revealed that CDC42 interaction inhibitors altered skin vascular arborization patterns, which were even more obvious when examining flattened images (Figures 4A and S4B). AngioTool quantification revealed that ARN22089 had a dose-dependent effect on the number of observed junctions and endpoints (Figure 4B, top). These results were conserved between experimental animals (Figure S4C). In contrast, vemurafenib or linifanib had a minimal effect on the number of observed junctions and no effect on the number of observed endpoints (Figure 4B, top). Next we measured the size distribution of skin vessels after drug treatment. Vemurafenib did not have a significant effect on the distribution of capillaries (<10 μm) or arterioles/venules (10–45 μm) in the skin (Figure 4B, bottom). Linifanib-treated skin had fewer capillaries, while ARN22089 most significantly inhibited the accumulation of both capillaries and arterioles/venules in a dose-dependent manner (Figure 4B, bottom). ARN22089 did not have any gross effects on skin appearance (Figure S4A) but did have modest effects on the thickness of the superficial dermal layer as detected by VVG staining and standard H&E staining (Figure 4C), akin to what was observed in RhoJ-KO mice (Figure 1D).

To verify that the effects of ARN22089 on vascularization were not strain dependent, we harvested skin from NSG mice bearing tumors that had been treated with CDC42 inhibitors, vehicle, or vemurafenib. Skin was harvested from regions that were at a minimum 5 mm away from the implanted tumor. We observed that ARN22089 treatment at either 20 or 40 mg/kg BID (twice a day) inhibited the accumulation of vessel junctions and endpoints as measured by AngioTool (Figures S5A and S5B). Notably, vemurafenib did not significantly alter either the accumulation of vessel junctions or endpoints (Figure S5B), similar to what was observed in C57B6 skin.

Next, we examined how ARN22089, ARN25062, and vemurafenib affected vessels in VMOs, which are the tumor-free version of the VMT micro-physiological system used earlier. These comprise of human endothelial cells, pericytes, and fibroblasts but not tumor cells (Figure 4D). We observed gross inhibition of vessel structure formation in the VMOs that were treated with ARN22089 or ARN25062, but this was not apparent in those treated with vemurafenib or vehicle (Figure 4E). REAVER quantification of VMO images revealed that treatment with ARN22089 and ARN25062 did indeed induce vascular disruption, evidenced by a significant reduction in vessel length and the number of branchpoints (Figure 4F). In contrast, the vasculature was not significantly affected by vemurafenib treatment (Figure 4F).

To examine tissue toxicity, we next examined how ARN22089 affected the vascularization of other organs. ARN22089 treatment had no effect on mouse weight (Figure S6A). ARN22089 had no effect on blood vessel accumulation in the brain (Figure S6B), although PK (pharmacokinetics) data did reveal that ARN22089 could cross the blood-brain barrier to a small degree (Figure S6C). Intriguingly, we did observe an effect on the accumulation of vasculature in the colon (Figure S6D), and this was accompanied by a loss of villi (Figure S6D).

### CDC42 interaction inhibitors modulate skin vascularization in an RhoJ-dependent manner

**Figure 5:**
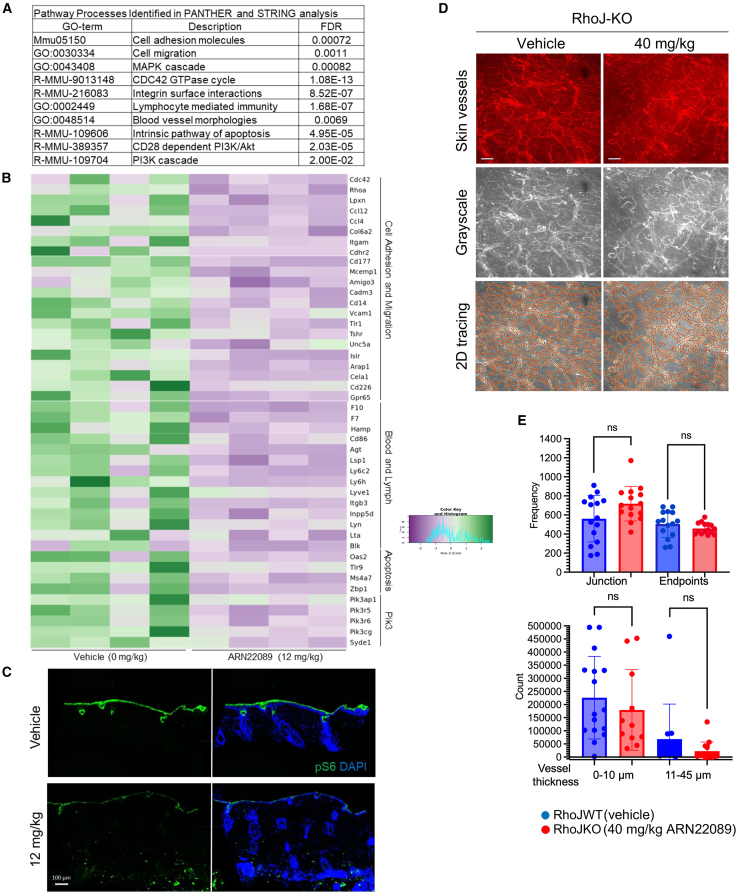
CDC42 inhibitors block skin angiogenesis in an RhoJ-dependent manner (A) Four mice were treated with 12 mg/kg ARN22089 or vehicle twice daily for 1 week, skin was harvested, and RNA was extracted from skin and subjected to bulk RNA sequencing (*n* = 4 animals per group). Differentially expressed genes (*p* < 0.01) were used to identify pathways in the STRING and PANTHER database that were downregulated in ARN22089-treated skin (pathways with a false discovery rate <0.002 are shown). (B) Heatmap showing significantly downregulated genes (at least 1.5-fold difference and *p* < 0.005) from the pathways identified in (A). (C) Immunofluorescence was used to detect the abundance of phosphorylated ribosomal S6 and S6 in skin treated with vehicle or 12 mg/kg ARN22089. DAPI was used to stain the nucleus. Scale bars, 100 μm. (D) Representative grayscale and AngioTool tracing of RhoJ-KO skin vessels (treated with vehicle or 40 mg/kg ARN22089). (E) Scatterplot showing the frequency of branches, termini, and vessel thickness as measured by the 2D AngioTool and local thickness (Fiji) software; n.s. no significant difference (*n* ≥ 3 per condition). Data representative as mean ± SD.

CDC42 interaction inhibitors block the interaction of both RhoJ and CDC42 with their downstream effectors,^46^ and both RhoJ-KO mice and CDC42 interaction inhibitor-treated mice had similar skin vessel arborization patterns. To better understand how ARN22089 modulates angiogenesis, we performed bulk RNA sequencing on WT skin treated with 12 mg/kg ARN22089 or vehicle twice daily for a week. We identified genes involved in CDC42 signaling and cell adhesion as genes downregulated after drug treatment, also noting that markers of cells that are carried within vascular compartments were also downregulated after drug treatment (Figure 5A; Tables S1, S2, and S3). Notable among these genes downregulated after drug treatment were the putative angiogenesis regulators CDC42,^59^ RhoA,^60^ and CCL4.^61^ Several genes involved in tumor angiogenesis/vasculogenesis, including leupaxin,^62^ CD177,^63^ and CCL12,^64^ were also downregulated in treated skin. Markers of lymphatic vasculature (Lyve1)^65^ and genes that mark cell types trafficked in lymphatics (lymphocytes, neutrophils, and macrophages) were also observed to be downregulated in drug-treated skin (Figures 5B; Table S4). We also observed that CD34 expression was reduced in ARN22089-treated animals (Figure 5B; Table S1). Several components of phosphatidylinositol 3-kinase signaling were also downregulated, consistent with earlier results that drug treatment affects S6 signaling.^46^ Consistent with these observations, we observed that S6 phosphorylation was reduced in ARN22089-treated skin samples (Figure 5C). The expression of Lyve1 and Cd45, a marker that marks many different immune cell types,^66^ was also reduced in treated skin (Figure S8C). We did not observe any gross skin toxicity in drug-treated animals, as the structure of the hair follicles was preserved (Figure 4C) and there were similar numbers of fibroblasts and endothelial cells in the skin of drug-treated and vehicle-treated mice (Figure S4D, top).

While we observed no difference in the number of endothelial cells or fibroblasts that accumulated in treated or untreated skin, we nonetheless identified changes in the expression of 16 genes involved in fibroblast and endothelial cell function (Figure S4E; Table S1). Among these changes were decreased expression of Col5a1, a gene that when mutated affects the accumulation of collagen in the dermis of patients with Ehlers Danlos.^67^ In addition, loss of expression of Fbnl1, a gene involved in elastic fiber formation,^68^ was seen. We observed decreased expression of Cd34 in the skin of ARN22089-treated animals, a result that we verified by immunofluorescence staining (Figure S8). Consistent with these observations, we identified a slight decrease in the thickness of the VVG-stained layer in the superficial dermis in drug-treated mice as compared to control mice (Figure 4C), similar to what was observed in RhoJ-KO mice (Figure 1D).

Previous work from our group demonstrated that ARN22089 and similar analogs blocked the interactions between both RhoJ and CDC42 and their downstream effectors. To examine whether the effects of ARN22089 on angiogenesis were RhoJ dependent, we treated WT and RhoJ-KO mice with ARN22089 and used the same approach as described in Figure 4 to measure changes in vascularization. Analysis of stacked and flattened images from the treated mice revealed no apparent differences in the vessel arborization patterns of RhoJ-KO mice that were treated with ARN22089 from baseline (Figure 5D). Similarly, we observed no differences in junctions or endpoints between RhoJ-KO-treated and untreated mice as measured by AngioTool (Figure 5E, top). There was no change in vessel size with ARN22089 treatment in RhoJ-KO mice (Figure 5E, bottom). 3D vessel analysis with neuTube similarly observed no differences in the number of branch nodes and end nodes between RhoJ-KO mice and RhoJ-KO mice treated with ARN22089 (Figures S1B and S1C).

**Figure 6:**
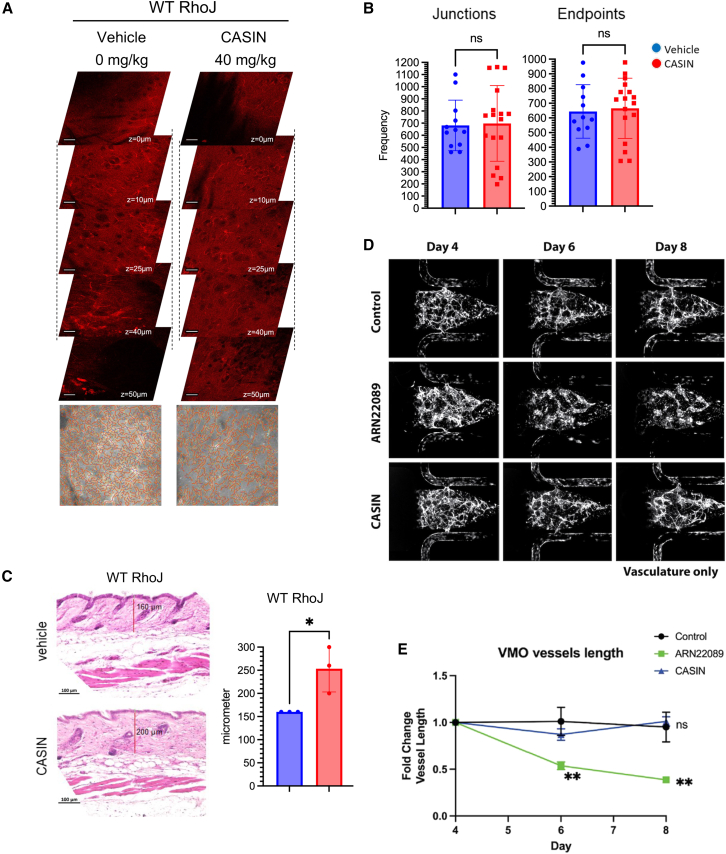
The selective CDC42 inhibitor CASIN does not affect vessel arborization in skin or vessel elongation *in vitro* (A) Representative stack images of skin vessels from wild-type mice treated with vehicle or 40 mg/kg CASIN intraperitoneally. Bottom of each stack includes the 2D AngioTool tracing of pixels in grayscale. Scale bar is 100 μm. (B) Scatterplot with bar graphs shows the frequency of branches, termini from flattened images using the 2D AngioTool. Dots in graphs correspond to analyzed image stacks (≥4 per stack). Unpaired two-tail t test was used to determine significance, *n* = 3 mice per condition. Data representative as mean ± SD. (C) Representative H&E staining of C57B6 skin obtained from CASIN or vehicle-treated mice is shown. The thickness of the dermis was quantified and compared between vehicle and drug-treated mice, *n* = 3 animals per group. Data representative as mean ± SD. (D) Representative fluorescent micrographs of dual-chamber VMO showing vasculature (grayscale), treated with control (vehicle only), ARN22089 (2 μM), or CASIN (2 μM) for 48 h starting on day 4. Media was refreshed on day 6, day 8, and day 10. (E) Quantification of VMO-associated vasculature showing fold change in vessel length and number of branchpoints compared to baseline. ^∗∗^*p* < 0.01. Data representative as mean ± SEM.

CASIN is a small molecule that selectively inhibits CDC42 GTP exchange without affecting RhoJ activation.^40^ To further examine whether CDC42 inhibition affected skin vasculature, we treated WT and RhoJ-KO mice with CASIN and quantified drug-induced changes in vascularization. Analysis of stacked and flattened images from treated mice revealed no apparent differences in vessel arborization patterns between CASIN-treated and vehicle-treated WT (Figures 6A and 6B) or RhoJ-KO mice (Figures S7A and S7B). Similarly, we observed that CASIN treatment did not affect the expression of Lyve1 (Figure S8A), a marker of lymphatic vessels. Published studies have indicated that CDC42-KO mice^69^ and CASIN-treated mice^42^ had an increase in dermal thickness when compared to control animals. We observe that CASIN treatment induces dermal thickening in both WT (Figure 6C) and RhoJ-KO mice (Figures S7C and S7D). CASIN treatment did not affect the expression of Cd34 (Figure S8B). Finally, we examine whether CASIN could inhibit vessel elongation in human-derived vascular organoids, as described in Figure 4. We observe that while ARN22089 inhibited vessel elongation in vascular organoids, CASIN did not (Figures 6D and 6E). Taken together, these results indicate that CDC42 interaction inhibitors block vascularization via a mechanism that is RhoJ dependent.

## Discussion

While many anti-angiogenic agents have been identified to treat cancer and other conditions, these agents have significant and frequent side effects,^70^^,^^71^ which preclude their use in genetic conditions that may arise in early life or those that would require chronic treatment, such as inflammatory skin disease. Moreover, the effects of VEGF inhibitors, the best-characterized anti-vascular agents, are transient with normal vessel structures returning even after 1 or 2 days of treatment cessation.^72^^,^^73^ To develop better agents, one needs to be able to quantify and compare the effects of drugs on vasculature in three dimensions. To accomplish this goal, we integrate tissue clearing with semi-automated vessel tracing and apply this approach to quantify the effect of anti-vascular agents *in vivo*. The straightforward 3D quantitative approach (neuTube) presented, previously applied to trace neurons, provides more insight into vascular structure than approaches where 3D images are flattened into 2D (AngioTool).

RhoJ selectively regulates tumor angiogenesis^37^ and retinal vascular angiogenesis during early development, a phenotype that resolves during adulthood.^35^^,^^36^ RhoJ does play a role in adult tissue homeostasis as RhoJ-KO mice have defective wound healing,^47^ although it is not clear whether this phenotype is a consequence of modulating the skin vasculature. We applied our quantitative imaging approach to show that RhoJ-KO mice had fewer small vessels (capillaries) in adult skin (Figure 1C), although RhoJ deletion did not affect the number of endothelial cells and fibroblasts (Figure 1E). While RhoJ deletion affected the vascularization of normal skin, it did not grossly affect skin structure, as the epidermis was intact and hair follicles were observed in RhoJ-KO mouse skin (Figure 1D). In contrast to the skin of RhoJ-KO mice, CDC42-KO mouse skin is characterized by epidermal thickening without reported changes to the vasculature.^69^ This suggests that these two GTPases play non-overlapping roles in skin homeostasis.

To further explore the efficacy of CDC42 interaction inhibitors as anti-angiogenesis agents, we examined the effect of these agents on the skin. ARN22089 treatment induced the downregulation of genes involved in cell migration and adhesion, as well as other genes known to play a role in angiogenesis (Figures 5A and 5B). It is notable that ARN22089 treatment downregulated the expression of Lyve1, a marker of lymphatic vasculature (Figure S8A). Future work will decipher whether this is a result of modulating angiogenesis with subsequent effects on lymphatics. In addition to its anti-angiogenesis effects, we observed that drug treatment downregulated the expression of genes such as collagen and fibulin, which impact dermal thickness. ARN22089-treated mice had decreased dermal thickness and collagen deposition as measured by VVG staining (Figure 4C) and reduced expression of Cd34 (Figure S8B). The anti-vascular activity of ARN22089 required the presence of functional RhoJ as ARN22089 did not inhibit angiogenesis in RhoJ-KO mouse skin (Figures 5D and 5E). ARN22089 had no effect on brain vasculature or mouse weight but did affect the vasculature in the colon (Figures S6B and S6D), indicating the broad anti-vascular activity of these compounds.

It is known that melanoma tumors are minimally responsive to anti-angiogenesis agents^6^ and tumor vessels can rapidly regrow after the cessation of VEGF-targeted therapies,^72^^,^^73^^,^^74^ with VEGF inhibitors having proclivity for targeting vessels at the periphery of the tumor rather than those at the center.^17^ VEGF inhibitors did not affect the number of branches and endpoints in skin, even though they did have some effects on the accumulation of skin capillaries (Figure 4B). The relatively modest effect of VEGF inhibitors on skin vascularization is consistent with their lack of efficacy in treating skin tumors. We did test the ability of one established melanoma therapy (vemurafenib) to inhibit tumor growth and angiogenesis. Vemurafenib treatment normalized vessel tortuosity and decreased vessel numbers in tumors *in vivo* (Figures 2C and 2D) and inhibited vessel elongation in VMTs *in vitro* (Figures 3E–3G). Unfortunately, we were unable to compare the efficacy of immunotherapy in the tumor models studied here as they lack a functional immune system.

CDC42 interaction inhibitors, while having similar growth inhibitory effects as vemurafenib on tumors *in vivo*, had profound differences in their anti-angiogenesis properties, revealed through the studies presented here in skin and *in vitro* vascular organoids. ARN22089-treated skin had fewer capillaries and arterioles (Figure 4B), consistent with the broad effects of ARN22089 and similar analogs on tumor vessels that was observed (Figure S2D). ARN22089 also affected the number of observed endpoints and vascular branches, suggesting that it inhibits vascularization by a different mechanism than either vemurafenib or linifanib in skin (Figure 4B). In contrast, vemurafenib (Figures 4A and 4B) did not affect skin vascularization. While ARN22089 could inhibit vessel elongation *in vitro* in vascular organoids, vemurafenib (Figures 4E and 4F) did not have any effect on vessel elongation in these models. As ARN220989 inhibited vascular elongation and not cell viability *in vitro*, the observed *in vivo* effects are unlikely to be secondary to endothelial cell toxicity.

One particularly intriguing observation was the distinct phenotypes observed with CASIN or ARN22089. CASIN, a selective CDC42 inhibitor, had no effect on skin vasculature (Figures 6A and 6B), induced dermal thickening (Figure 6C), and did not affect Cd34 or Lyve1 expression in skin (Figures S8A and S8B). Neither of these phenotypes is RhoJ dependent (Figure S7). We also demonstrated that CASIN did not affect vessel elongation in vascular organoids (Figures 6D and 6E). Taken together, these results indicate that RhoJ plays a specific role in skin vascularization, which is distinct from CDC42. CDC42 and RhoJ play antagonistic roles in fibroblasts, with CDC42 inhibiting skin thickening and RhoJ promoting skin thickening. It is clear that while CASIN and other analogs that target CDC42 GTPases may have promising anti-aging properties, they would have little efficacy in preventing angiogenesis that is commonly observed in inherited and inflammatory skin disease.

In summary, the work presented here provides evidence that small molecules that target RhoJ signaling, as compared to those that only target CDC42, have potent anti-vascular effects in skin. The relative lack of systemic toxicity (mice maintained a healthy body weight) and CNS toxicity as compared with the rather specific effects of these agents on the skin and colon suggests that, at a minimum, they could be used as topical agents to prevent vascularization of skin tumors or to inhibit vessel accumulation in the context of inherited vascular disorders or skin inflammatory diseases. Notably, the anti-tumor activity of these agents was on par with mitogen-activated protein kinase inhibitors, justifying further exploration of these drugs as systemic anti-cancer agents. While the studies here indicate that CDC42 GTPases have non-redundant roles in angiogenesis, they still may have redundant roles in other cellular processes, such as cell migration/immune cell function, which are not tested in this study. Deciphering the precise roles of these CDC42 GTPases in different processes using genetic and pharmacologic tools will be a topic for future work.

### Limitations of the study

As current *ex vivo* human skin models lack a perfused vascular supply, we were unable to test whether these agents could block vascularization of intact human skin. Moreover, we were unable to directly compare the efficacy of different CDC42 inhibitor formulations on skin as oral formulations were not available for all analogs and topical formulations have not been developed for any of them. Direct measurement of CDC42 activity in endothelial cells in tissue was not possible at the current time secondary to the lack of precise *in vivo* reporters to measure CDC42 activation, which is currently under development.

## Resource availability

### Lead contact

Further information and any requests should be directed to and will be fulfilled by the lead contact, Professor Anand K. Ganesan (aganesan@hs.uci.edu).

### Materials availability

This study did not generate new unique reagents.

### Data and code availability

RNA-seq data have been deposited at GEO: GSE298488 and are publicly available. The accession number is listed in the key resources table. This paper does not report any original code. Any additional information required to reanalyze the data reported in this work is available from the lead contact upon request.

## Acknowledgments

This work was supported by CA-244571 from the 10.13039/100000054National Cancer Institute and S10OD028698. This study was made possible in part through access to the Optical Biology Core Facility, a shared resource supported by the Cancer Center Support Grant (CA-62203) and through access to the Imaging Core of UCI Skin (AR-075047) at the 10.13039/100005595University of California, Irvine, United States. In addition, UC Irvine Clinical Innovation Incubator, United States, provided support for the work comparing CASIN and CDC42 interaction inhibitors. M.D.V. thanks the 10.13039/501100005010Italian Association for Cancer Research (AIRC), Italy, for financial support under the Investigator Grant scheme (IG 25003 and IG 30631).

## Author contributions

Conceptualization, A.K.G., C.C.W.H., M.D.V., B.C., and L.M.V.; funding acquisition and project administration, A.K.G.; data curation, L.M.V., S.H., J.S., S.M.B., N.B., M.S., R.B., and A.A.; formal analysis, L.M.V., S.H., J.S., S.M.B., N.B., M.S., R.B., A.A., and V.S.H.K.; investigation, L.M.V., S.H., J.S., N.S., D.F.X., and T.N.; writing – original draft, L.M.V. and A.K.G.; writing – review and editing, L.M.V., A.K.G., C.C.W.H., M.D.V., B.C., D.F.X., S.H., and J.S.; methodology, N.B., S.M.B., M.S., R.B., A.A., T.N., N.S., D.F.X., R.P., V.S.H.K., and S.D.L.

## Declaration of interests

C.C.W.H. is a founder of, and has an equity interest in, Aracari Biosciences, Inc., which is commercializing the VMT model. The small molecules used in this study are a joint invention of investigators at the Italian Institute of Technology and UC Irvine. All work is with the full knowledge and approval of the UCI Conflict of Interest Oversight Committee.

## STAR★Methods

### Key resources table

**Table:** 

REAGENT or RESOURCE	SOURCE	IDENTIFIER
**Antibodies**		

Monoclonal CD31 (PECAM-1) Brillian Violet 421	eBioscience	Cat# 404-0311-82; RRID:AB_2929070
Monoclonal CD140a (PDGFRA) PE	eBioscience	Cat# 12-1401-81; RRID:AB_657615
Fixable Viability Dye eFlour 780	eBioscience	Cat# 65-0865-14
Mouse CD45	R&D	Cat# MAB114; RRID: AB_357485
Monoclonal Lyve1 (ALY7)	eBioscience	Cat# 14-0443-82; RRID:AB_1633414
Phospho-S6 Ribosomal Protein (Ser235/236) −488	Cell Signaling	Cat# 4803; RRID: AB_916158
S6 Ribosomal Protein −488	Cell Signaling	Cat# 5317; RRID:AB_10694920
Anti-mouse CD31	eBiosciences	Cat# 553370; RRID:AB_394816
Monoclonal CD34 (RAM34)	eBioscience	Cat# 14034182; RRID:AB_467210
Lectin (LEL, TL) DyLight 649	Vector Lab	Cat# DL-1178-1

**Biological samples**		

BRAFV600K,PTPN11N58S	National Cancer Institute	563396-261-R

**Chemicals, peptides, and recombinant proteins**		

Linifanib (ABT869)	MedChemExpress	Cat# HY-50751
Vemurafenib (PLX4032)	SellekChem	Cat# S1267
CASIN	MedChemExpress	Cat# HY-12874

**Deposited data**		

RNA sequence data	This paper	GEO: GSE298488

**Experimental models: Cell lines**		

Human: WM3248	Coriell Medical Institute	Cat# WC00081; RRID:CVCL_6798
Human: A375	ATCC	Cat# CRL-1619; RRID:CVCL_0132
Human: cell-derived endothelial cells	Lab of Hughes	Hachey et al.^56^
Human: Lung Fibroblasts	Lonza	Cat# CC-2512

**Experimental models: Organisms/strains**		

*NOD.Cg-Prkdc*^*Scid*^	Jackson	RRID:IMSR_JAX:005557
*C57BL/6J*	Jackson	RRID:IMSR_JAX:000664

**Software and algorithms**		

ImageJ	National Institute of Health Bethesda USA	https://imagej.nih.gov/ij/
AngioTool	National Cancer Institute	Zudire et al.^51^
neuTube	Howard Hughes Medical Institute	Feng L et al.^52^
REAVER	BSD 3.0 open source	Corliss et al.^58^
PANTHER	https://pantherdb.org/	Mi H et al.^75^
STRINGv12	https://string-db.org/	Szklarczyk D et al.^76^
RUVSeq	www.bioconductor.org/	Risso D et al.^77^
Samtools/1.15.1	Lab of Trapnell	Li H et al.^78^
Prism 10.4.1	GraphPad	www.graphpad.com/
HISAT2 v2.2.1	Lab of Trapnell	Kim et al.^79^
FlowJo	BD Bioscience	https://www.flowjo.com/
MATLAB	MathWorks	www.mathworks.com/products/matlab.html

### Experimental model and study participant details

#### Cell line

All cell lines were maintained according to the manufacturers. Details of cell culture are described in method details below.

#### Mice

All animal experiments were approved by the UC Irvine Institutional Animal Care and Use Committee (IACUC). Details of experimental procedure in mice are described in method details below.

### Method details

#### *In vivo* skin and tumor experiments

All animal experiments were approved by the UC Irvine Institutional Animal Care and Use Committee (IACUC) (AUP-20-161). C57BL6 mice and RhoJ KO mice in the C57B6 background were used in studies examining skin, colon, and brain vascularization while NOD.Cg-*Prkdcscid IL2rgtm1Wj*/SzJ (NSG) mice were used for the patient-derived xenograft experiments. An equal number of male and female mice were used in treatment with inhibitors. For skin treatment, wild-type or RhoJ KO C57BL6 mice were treated with inhibitors by oral gavage twice daily for one week. For PDX tumor experiments, tumors were maintained by passaging into NSG mice before treatment. Inoculation of tumors in NSG mice was performed as previously described.^55^ When tumors reached ∼150–200 mm^3^, mice were treated by oral gavage twice daily with 20 or 40 mg/kg ARN22089 or with vemurafenib at 10 or 25 mg/kg, or with linifanib 10 mg/kg. Tail-vein injection was done once a day at 10 mg/kg ARN22089. Intraperitoneal injection of CASIN was performed at a dose of 40 mg/kg once a day for a week. All tumor experiments involved treating animals for two weeks while skin experiments involved treatment for 1 week.

#### Cardiac perfusion and tissue collection

At time of harvest, mice were infused with lectin-DyLight-649 (200 μL, 25% lectin-DyLight and 75% PBS) via tail vein injection to label endothelial cells. One hour later, mice were euthanized, and cardiac perfusion with 50 mL saline followed by 4% methanol-free paraformaldehyde (PFA) was performed as described previously.^49^ For skin, the fur was shaved and depilated and dorsal skin was removed from the back prior to perfusion and tissue collection. Tumors were removed from the flanks of the dorsal region. Brain, kidney, intestines, livers, hearts, and tissues were placed in 4% PFA for 24–48 h and then placed in 1xPBS at 4°C for subsequent tissue clearing.

#### Tissue clearing and imaging analysis

A modified iDISCO protocol was used to clear tissues. Tissues were dehydrated in graded series (20, 40, 60, 80, 100, 100%) of methanol in water for 48 h at RT. Samples were incubated with 66% dichloromethane (DCM) and 33% methanol for 48 h at RT. Next, samples were washed twice for 15 min each with DCM and placed in dibenzyl ether (DBE) for storage and used as a medium for imaging. The Leica TCS SP8 X instrument was used to image tissues and processed with a LAS X Navigator suite. The sample was placed on a makeshift holder attached to a microscope slide and filled with DBE; a coverslip was placed over the sample, making sure the sample and liquid touches the coverslip. Images were captured using an HC PL FLOUTAR 10x/0.30 objective. Image dimension: 1024 × 1024 pixel dimension and 1107.14 μm by 1107.14 μm, 5 μm per z stack. Vascular fluorescence was detected by scanning for signals in the DyLight649 spectrum (670–708 nm). For image analysis, z stack images were opened with Fiji ImageJ, and color images were converted to black background and white pixels using color-split and saved as a tiff file either in three-dimenasional (3D) or compressed into 2D image using z stack projection. 3D tiff files were analyzed using neuTube software,^52^ while 2D images were analyzed using AngioTool software.^51^ Both automated and manual tracing were performed on Tiff images in neuTube. Automated tracing was applied initially, with manual curation as needed in cases of low contrast between vessels and background and where large vessels cannot be correctly auto-traced. A custom MATLAB script was used to extract the number of branch and endpoints, vessel length, vessel diameter, and tortuosity in the neuTube SWC format files (individual nodes with x, y, z coordinates, radius and node connectivity). In the SWC format, vessel structures are simplified into spherical nodes with the radius of the vessel at a given location. Branch points (green nodes in images) appear where branches occur, and endpoints (yellow nodes in images) appear at the end of a vessel segment (all other nodes are red in images). A vessel segment is defined as a series of nodes that are bounded by two branch points or one branchpoint and one endpoint. The length of a vessel segment is defined as the distance between its pair of boundary points. The diameter of the vessel is calculated using the radius of the nodes. Tortuosity is calculated by dividing the chord (distance of two ends of a vessel) over the vessel length; value <0.5 is highly tortuous. Parameters for AngioTool analysis were set the same for all images analyzed. Same 2D images, which were used in AngioTool, were used to determine the width of the vessel thickness using Local Thickness (masked, calibrated, and silenced) plugin on Fiji software; width <10 μm represents veins and capillaries and between 11 and 45 μm represents arterioles in the skin.^80^ For NSG skin stack images, the first and last few images in the stacks were removed to reduce background noise; all stacks from C57BL6 skin were analyzed. For image analysis >3 tumors or skin tissues were cleared, imaged and analyzed.

#### Statistics

GraphPad Prism software was used to generate graphs and perform statistical significance, using one- and two-way ANOVA test and unpaired T-test. One-way ANOVA and unpaired T-test were applied on vessel analysis and two-way ANOVA test was used to determine significance for tumor growth curves. Custom R script was used to convert pixel value to micrometer for diameter and distance; it was also used to calculate the frequency of branch nodes, end nodes, number of vessels and branching, and tortuosity.

#### Microfluidic device fabrication

Microfluidic device fabrication and loading have been previously described.^57^^,^^81^ In summary, a custom polyurethane master mold was created using a two-part polyurethane liquid plastic (Smooth Cast 310, Smooth-On Inc.). Subsequently, a polydimethylsiloxane (PDMS) layer was replicated from this master mold, and holes were punched to create inlets and outlets. The platform was assembled in two stages: first, the PDMS layer was chemically glued and subjected to 2 min of oxygen plasma treatment to affix it to the bottom of a bottomless 96-well plate (Greiner). Following this, a 150 μm thin transparent membrane was bonded to the bottom of the PDMS device layer through an additional 2-min treatment with oxygen plasma. The fully assembled platform was then placed in a 60°C oven overnight, covered with a standard 96-well plate polystyrene lid, and sterilized using UV light for 30 min before cell loading.

#### Cell culture and microfluidic device loading

To establish the vascular chamber, normal human lung fibroblasts and ECFC-ECs (endothelial colony forming cell endothelial cells^56^^,^^57^) or HUVECs (human umbilical vein endothelial cells) were harvested and resuspended in fibrinogen solution at a concentration of 3×10^6^ cells/mL and 7×10^6^ cells/mL, respectively. For VMT, A375 and WM3248 melanoma cells were introduced into the tumor chamber at a concentration of 1 × 10^5^ to 2 × 10^5^ cells/mL fibrinogen solution. Fibrinogen solution was prepared by dissolving 70% clottable bovine fibrinogen (Sigma- Aldrich) in EBM2 basal media (Lonza) to a final concentration of 5 mg/mL. The cell-matrix suspension was mixed with thrombin (50 U/mL, Sigma-Aldrich) at a concentration of 3 U/mL, quickly seeded into the microtissue chambers, and allowed to polymerize in a 37°C incubator for 15 min. Laminin (1 mg/mL, LifeTechnologies) was then introduced into the microfluidic channels through medium inlets and incubated at 37°C for an additional 15 min. After incubation, culture medium (EGM-2, Lonza) was introduced into the microfluidic channels and medium wells. The medium was changed every other day, and the hydrostatic pressure head re-established daily to maintain interstitial flow.

#### Drug treatment in the VMO and VMT

Following a culture period of 4–5 days to facilitate the development of a perfused vasculature within each VMO or VMT, the culture medium was replaced with a medium containing the specified drug concentrations. Drugs were administered to the microtissues through the newly formed vascular bed via gravity-driven flow. Specifically, ARN25062, ARN22089, and vemurafenib were used at a 2 μM dose. A375 VMT, WM3248 VMT, and VMO were randomly assigned to one of four conditions: control (vehicle only), 2 μM ARN25062, 2 μM ARN22089, 2 μM vemurafenib, or 2 μM CASIN. For experiments with two vascular side chambers, the left chamber received 2 μM ARN22089, while the right chamber served as control (vehicle only). Both VMO and VMT underwent a 48-h treatment period, with complete medium replacement every 48 h. Fluorescent micrographs of VMT were captured every 48 h for 6 days post-treatment, and the quantification of tumor and vasculature growth was performed.

#### Fluorescence imaging and analyses of VMT/VMO

Fluorescence images were acquired with a Biotek Li- onheart fluorescent inverted microscope using automated acquisition and standard 10x air ob-jective. To test vessel perfusion, 25 μg/mL FITC- or rhodamine-conjugated 70 kDa dextran was added to the medium inlet prior to treatment. For quantifying vessel length in VMOs and VMTs, Rapid Editable Analysis of Vessel Elements Routine (REAVER) software^58^ (MATLAB) was employed. ImageJ software (National Institutes of Health) was utilized to determine the total fluorescence intensity (mean gray value) for each tumor image, providing a measure of tumor growth. Normalization to baseline was performed for each chamber. In VMTs, tumor growth was quantified by measuring the total fluorescence intensity in the color channel representing the tumor cells. This measurement accounted for both the area and depth of individual tumors, considering that thicker areas appear brighter. Any image adjustments made were applied uniformly to ensure consistency across all images in the experimental group.

#### Tissue histology and immunohistochemistry

Tissues were fixed in 4% formaldehyde and washed in 1xPBS. For embedding, tissues were incubated in 65% ethanol for 30 min at RT and kept in 70% ethanol before sending the samples to Experimental Tissue Resource (ETR) at UCI for embedding, VVG^82^ and H&E staining.^83^ Anti-Cd31 was used in embedded tissue (rat anti-mouse Cd31, Fisher Scientific). For immunofluorescence analysis, tissues were snap frozen in OCT and stored in −80°C for later sectioning on a cryostat. Primary antibodies used include: Cd34 and Lyve1(eBioscience), Cd45 (R&D); Alexa 488 (Life Technology). Cell Signaling conjugated pS6 and S6 (Alexa Fluor 488, #4803, #5317).

#### Flow cytometry

Back skin from mice was first shaved and depilated. A large surface area (6 cm × 3 cm) of the skin on the back was cut and fat removed. The skin was minced, and digest buffer added (RPMI without Ca/Mg (no FBS/EDTA), 23.2 mM HEPES and 2.32 mM sodium pyruvate, 0.25 mg/mL Liberase, 46.4 unit DNase) and incubated for 1.5 h at 37°C shaking. Spleen samples were removed from the same animals, minced and RPMI added to minced tissue. All samples were filtered with 70 micrometer cell strainer, and washed with FAC buffer (5% FBS, 2 mM EDTA, 1% non-essential amino acids, 3.9x beta-mercapthoethanol (from 1000x)) twice and spun at 18.0 xg for 10 min 4°C. Skin samples were blocked with TruStain FcX PLUS and spleen samples with Fc block, both, at 1:100 for 15 min at 4°C. All samples washed twice with FAC buffer and incubated in primary Cd31 (1:50, brilliant violet 421 Invitrogen 404031182), PDGFRα (1:100, APA5 Life Technology 12140181), viability (1:200, eBioscience dyefluor 780 65086514) in FAC buffer for 30 min at 4°C nutating. Samples were then washed 2x and fixed with 1% formaldehyde for 15 min and washed twice again. Samples were resuspended with 400 μL FAC buffer and ran on the BD Fortessa X20 Flow cytometer. Compensation and FMO for both skin and spleen were run before running the experimental samples. Samples were analyzed using FlowJo.

#### Quantification of brain levels of ARN22089 following oral administration

Animals were treated with ARN22089 at 10 mg/Kg by oral administration, as described.^45^ Mouse brains were collected at 1, 2, 4 and 8 h after administration. The brains were homogenized and, following protein precipitation with acetonitrile, the amount of ARN22089 was quantified by LC-MS/MS as described.^45^

#### Bulk RNA sequencing and pathway analysis

A small piece (approximately 3 × 3 cm section) of the skin from a mouse was removed and placed in Qiagen buffer RLT (plus beta-mercaptoethanol). A Precellys Homogenizer was used to homogenize the skin sample in Precellys beads. RNeasy kit (Qiagen) was used to extract RNA, with DNase I digestion step. The RNA samples (*n* = 4) were sent to the Genomic Core Facility at UCI for library construction and sequencing. Sequencing reads were aligned and feature count to the mouse reference UCSC/mm10 (ERCC spike-in reference was concatenated with the mouse genomic and transcript)^84^ with HISAT2 v2.2.1^79^ and samtools/1.15.1^78^^,^^85^ to generate the feature count. Count matrix was generated in R programming and RUVSeq^77^ package was used to normalize (RUVr, residuals) RNA-seq data and determine differential expression. List of differential genes (vehicle vs. ARN22089 (12 mg/kg)) was used to run functional processes on PANTHER^75^^,^^86^ (*p* < 0.09) and STRINGv12^76^ (*p* < 0.009) to determine pathway analysis^87^ and gene ontology (Tables S1, S2, S3, and S4). Heatmap was generated using R programming.

### Quantification and statistical analysis

Any quantification and statistical analysis that were applied in the experiments are described in method details, as well as in the main and supplemental figure legends.

## Supplemental information

Document S1. Figures S1–S8

Table S1. Excel file containing additional data too large to fit in a PDF, related to Figures 5A, 5B, and S4E Complete list of differentially expressed genes. Analysis of bulk RNAseq data was performed using the RUV seq package in R.

Table S2. Excel file containing additional data too large to fit in a PDF, related to Figures 5A and 5B List of unique differentially expressed genes in Tab 1 with p < 0.09 that were then mapped to PANTHER pathways.

Table S3. Excel file containing additional data too large to fit in a PDF, related to Figures 5A and 5B Overrepresented pathways when genes in Tab2 were subjected to Panther pathway and reactome analysis.

Table S4. Excel file containing additional data too large to fit in a PDF, related to Figure 5B Unique genes with p < 0.009 from Tab1 were annotated in STRING to identify overrepresented functional processes.
