# Supplementary material for: CDC42-effector interaction inhibitors alter patterns of vessel arborization in skin and tumors in vivo
Source: iScience. 2025 Jul 14;28(7):112971. doi: 10.1016/j.isci.2025.112971 (PMC12432855; doi:10.1016/j.isci.2025.112971)
Supplement: Document S1. Figures S1–S8 [file mmc1.pdf]

iScience, Volume *28*

Supplemental information

CDC42-effector interaction inhibitors alter patterns of vessel arborization in skin and tumors *in vivo*

Linh M. Vuong, Stephanie Hachey, Jessica Shiu, Danny F. Xie, Noel Salvador, Nicoletta Brindani, Sine Mandrup Bertozzi, Maria Summa, Rosalia Bertorelli, Andrea Armirotti, Rachel Pham, Vance S.H. Ku, Swara D. Limbekar, Terry Nguyen, Bernard Choi, Christopher C.W. Hughes, Marco De Vivo, and Anand K. Ganesan

Figure S1

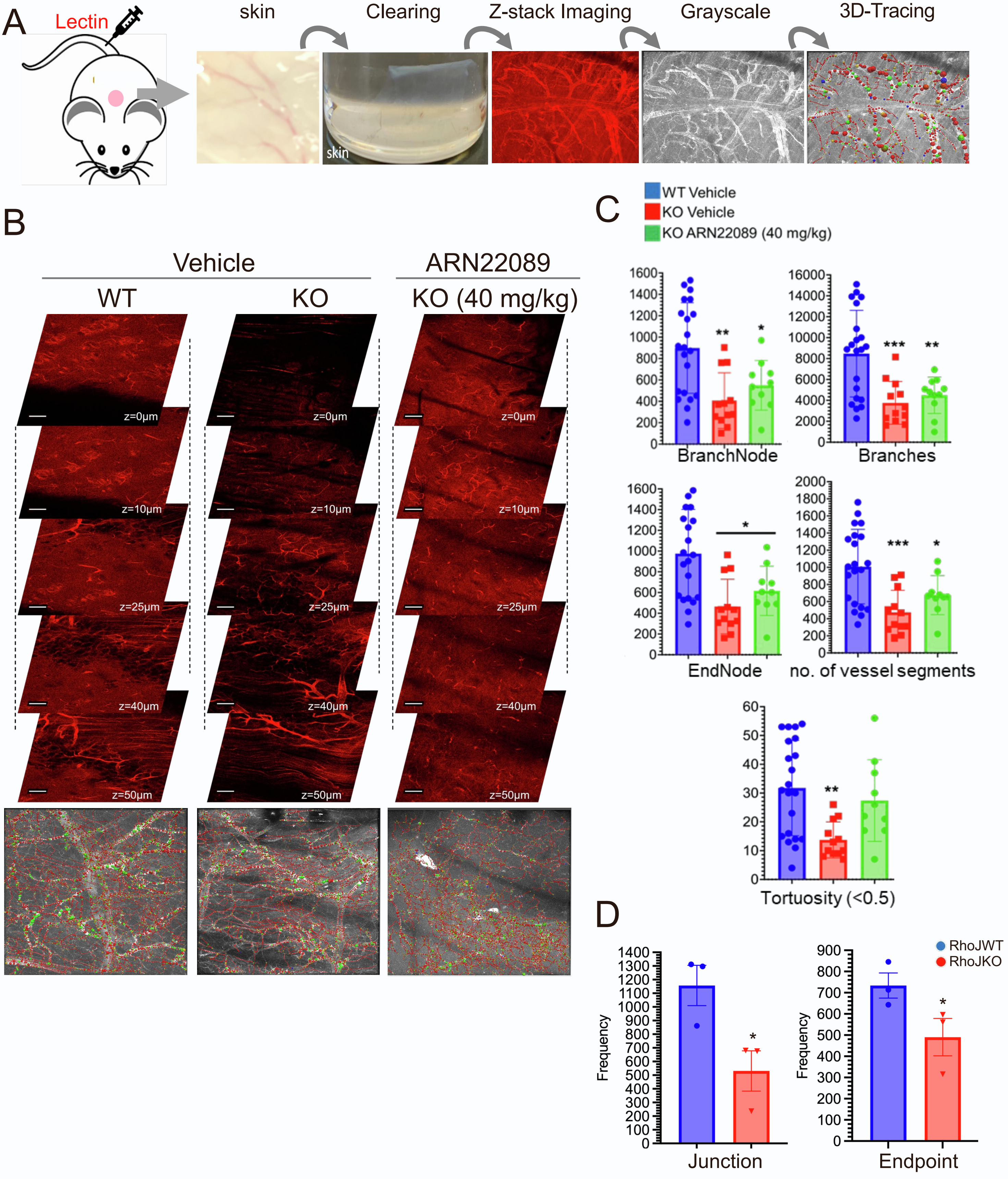
A
Lectin
skin
Clearing
Z-stack Imaging
Grayscale
3D-Tracing
skin
B
Vehicle
ARN22089
WT
KO
KO (40 mg/kg)
z=0μm
z=10μm
z=25μm
z=40μm
z=50μm
C
WT Vehicle
KO Vehicle
KO ARN22089 (40 mg/kg)
BranchNode
Branches
EndNode
no. of vessel segments
Tortuosity (<0.5)
D
RhoJWT
RhoJKO
Frequency
Junction
Endpoint

Figure S1. RhoJ deletion modulates skin vascular architecture, related to Figure 1. (A) Diagram of optical tissue clearing and vessel analysis. Each image (1.107 x 1.107 mm; 1024 x 1024 pixels) has 50-100 z-stacks (5 µm/stack). Z-stacks are compressed into 3D a Tiff file, converted to grayscale, and analyzed with neuTube. Images were generated from three mice per group. (B) Representative images of skin vasculature from RhoJ wild type (WT) vs knockout (KO) [vehicle vs 40 mg/kg twice daily a week] mice. Fluorescence images of lectin labeled structures were obtained, converted to grayscale, and traced with neuTube. Scale bar is 100 µm. (C) Scatter plot with bar graphs show the statistical quantification of vessel parameters (number of branch/end points and branching, and tortuosity) after 3D vessel tracing. Each dot corresponds to an analyzed image ($\geq$ 4 images per condition). Unpaired 2-tail t-test was used to determine p-value. *,**,***p < 0.05, 0.01, 0.0005, vehicle vs drug treated tumors. $\geq$ 3 mice (WT or RhoJ knockout) were analyzed. Data are represented as mean$\pm$SD. (D) Data from Figure 1C were replotted, averaging the results compiled from images of each animal (each dot corresponds to an animal) (unpaired two-tail *p < 0.05). Data are represented as mean$\pm$SEM.

Figure S2

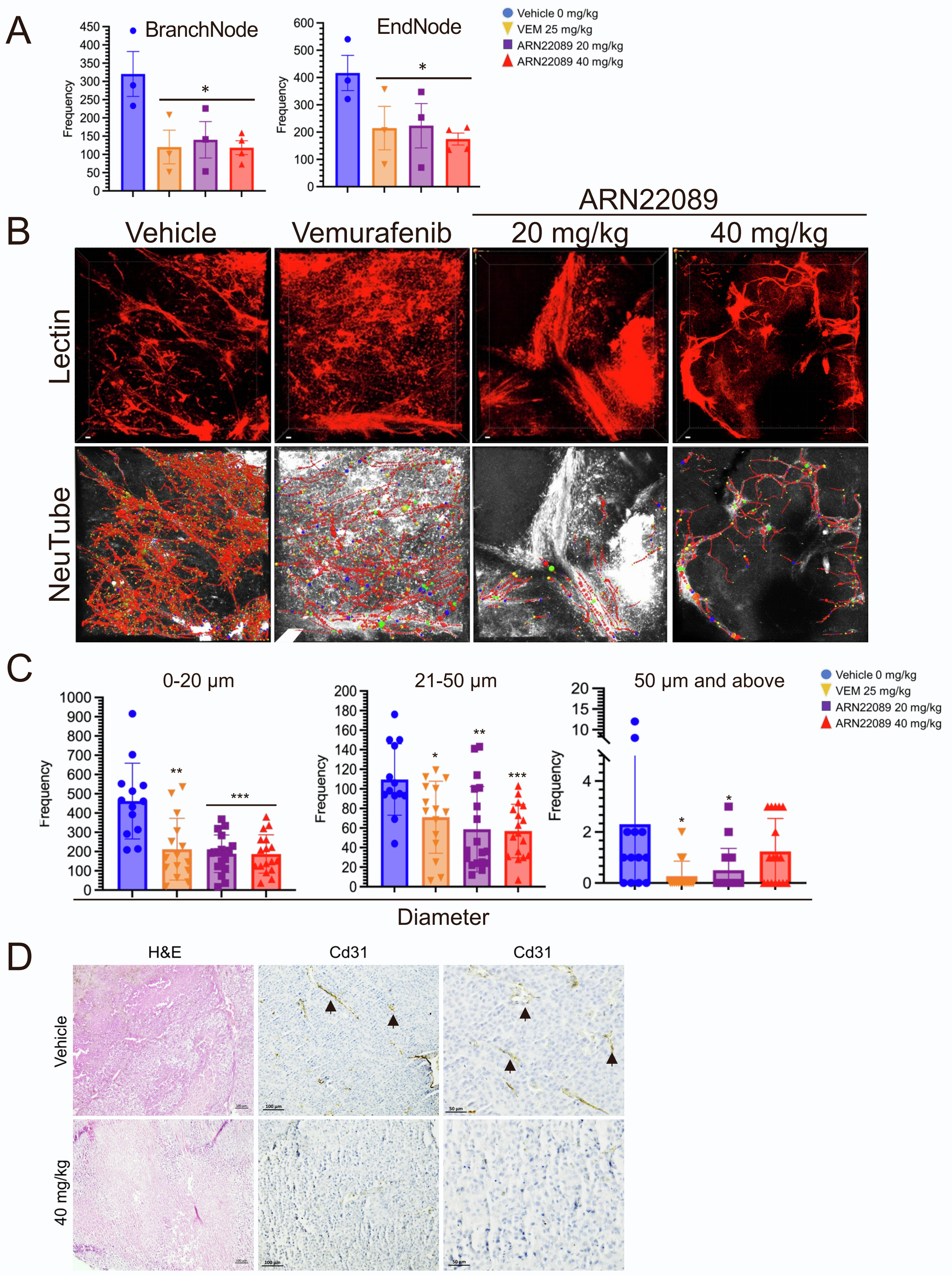

A
BranchNode
EndNode
Frequency
Vehicle 0 mg/kg
VEM 25 mg/kg
ARN22089 20 mg/kg
ARN22089 40 mg/kg
B
Vehicle
Vemurafenib
ARN22089
20 mg/kg
40 mg/kg
Lectin
NeuTube
C
0-20 μm
21-50 μm
50 μm and above
Frequency
Diameter
D
H&E
Cd31
Cd31
Vehicle
40 mg/kg

Figure S2. ARN22089 affects small and large tumor vessels, related to Figure 2. (A) Data from Figure 2D were replotted, averaging the results compiled from images of each animal (each dot corresponds to an animal) (unpaired two-tail *p < 0.05). Data are represented as mean±SEM. (B) Additional representative 3D images of cleared tumors showing vessels labeled from mice treated with the indicated inhibitors for two weeks. Images were converted to grayscale and saved as a tiff file for 3D tracing with neuTube as shown in figure 2. Scale bar = 100 μm. Each image (1.107 x 1.107 mm; 1024 x 1024 pixels) has 207 z-stacks (5 μm/stack). (C) Scatter bar plots showing additional vessel parameters, similar to those described in Figure 2D. The number of vessels in treated tumors that were <20 microns in diameter, between 21-50 microns in diameter, and >50 μm in diameter was plotted. Each dot corresponds to an individual stack, and 3 tumors were analyzed per group. *,**,***p < 0.05, 0.01, 0.0005, (vehicle vs treated tumor vessels). Data are represented as mean±SD. (D) Representative images of Cd31 staining of vehicle and ARN22089-treated tumors (representative images from two tumors of the three biological replicates performed). Arrowhead points to endothelial cells expressing Cd31.

Figure S3

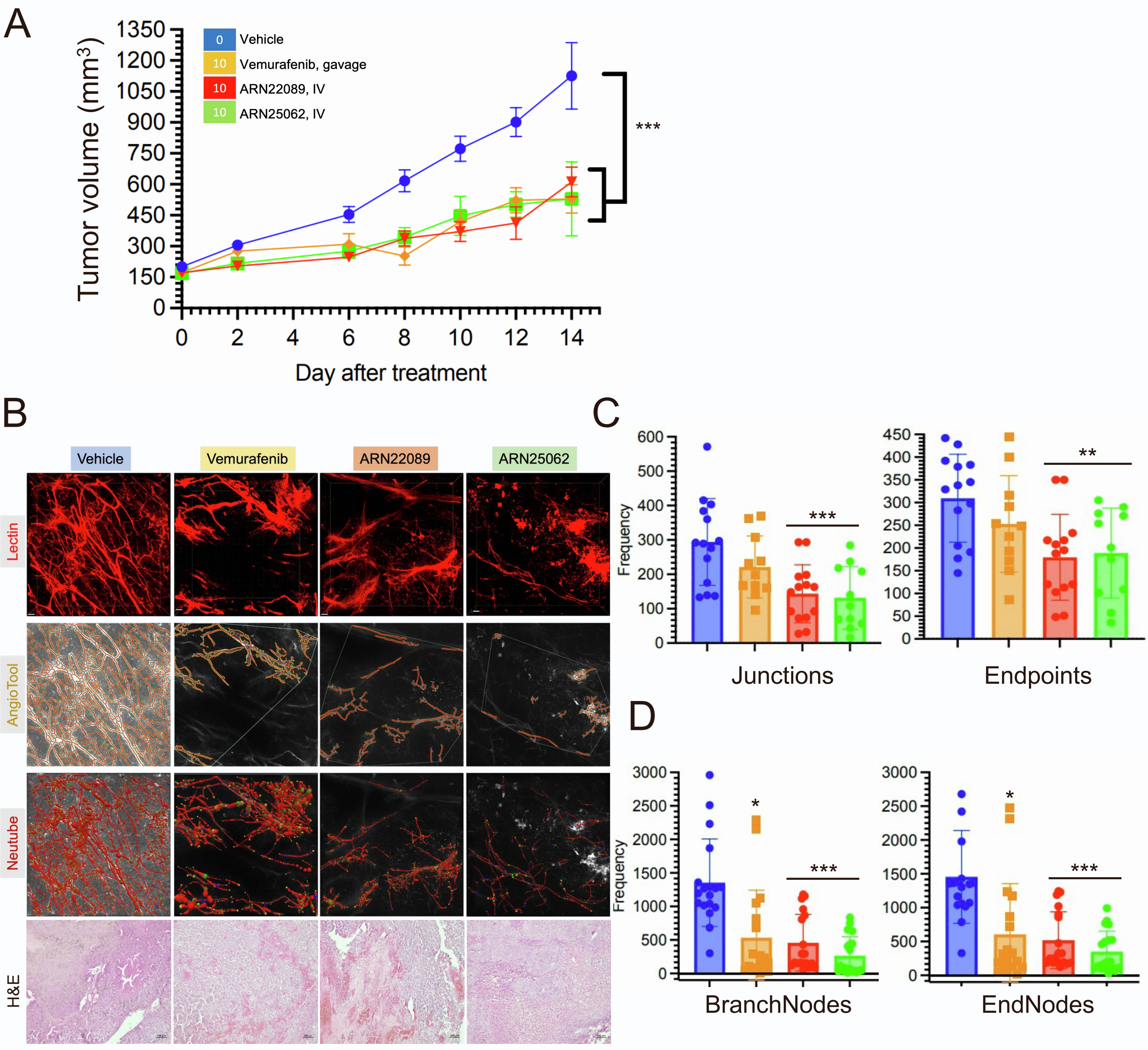
A
Tumor volume (mm^3)
1350
1200
1050
900
750
600
450
300
150
0
Day after treatment
0
2
4
6
8
10
12
14
0 Vehicle
10 Vemurafenib, gavage
10 ARN22089, IV
10 ARN25062, IV
***
B
Vehicle
Vemurafenib
ARN22089
ARN25062
Lectin
AngioTool
Neutube
H&E
C
Frequency
Junctions
Endpoints
***
**
D
Frequency
BranchNodes
EndNodes
*
***

Figure S3. ARN25062 disrupts tumor angiogenesis, related to Figure 2 and 3. (A) Growth curves of tumors treated with the indicated doses of ARN25062 and ARN22089 by IV were compared to tumors that were treated with vemurafenib 10 mg/kg BID for two weeks once daily. Data are represented as Mean±SEM, Two-way ANOVA test indicates significance between vehicle and treated mice for ARN22089, ARN25062 and Vemurafenib ***p < 0.0005. (B) Representative 3D images of cleared tumor showing vessels labeled with lectin DyLight from mice treated with indicated inhibitors. *Center*, images were converted to grayscale and saved as a tiff file for 2D tracing with AngioTool or 3D tracing with neuTube. *Bottom*, H&E staining of tumors. Scale bar = 50 µm. Each image (1.107 x 1.107 mm; 1024 x 1024 pixels) has 207 z-stacks (5 µm/stack) (C) Scatter plot with bars show the statistical quantifications from Angiotool tracing of vessel branching and endpoints. (D) Scatter plot with bars showing metrics obtained from neuTube tracing of vessel branching and endpoints. Unpaired 2-tail T-test applied in 2D and 3D analysis; **,***p < 0.01, 0.0005. ≥3 tumors per condition were analyzed, ≥ 4 images per condition. Data are represented as mean±SD.

Figure S4

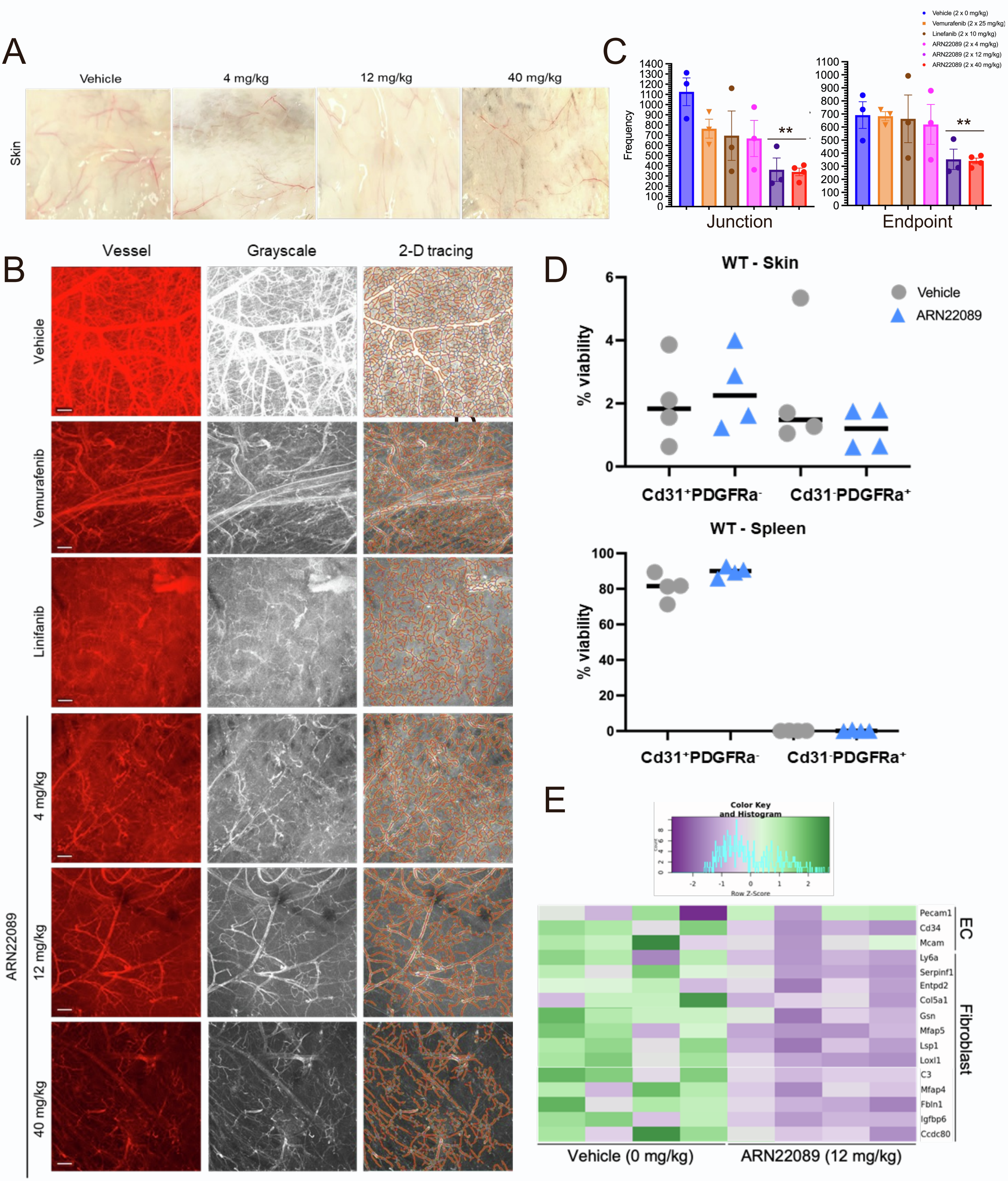

A
Vehicle
4 mg/kg
12 mg/kg
40 mg/kg
Skin
B
Vessel
Grayscale
2-D tracing
Vehicle
Vemurafenib
Linifanib
ARN22089
4 mg/kg
12 mg/kg
40 mg/kg
C
Frequency
Junction
Endpoint
**
Vehicle (2 x 0 mg/kg)
Vemurafenib (2 x 25 mg/kg)
Linefanib (2 x 10 mg/kg)
ARN22089 (2 x 4 mg/kg)
ARN22089 (2 x 12 mg/kg)
ARN22089 (2 x 40 mg/kg)
D
WT - Skin
% viability
Vehicle
ARN22089
Cd31+PDGFRa-
Cd31-PDGFRa+
WT - Spleen
E
Color Key and Histogram
Count
Row Z-Score
Pecam1
Cd34
Mcam
Ly6a
Serpinf1
Entpd2
Col5a1
Gsn
Mfap5
Lsp1
Loxl1
C3
Mfap4
Fbln1
Igfbp6
Ccdc80
EC
Fibroblast
Vehicle (0 mg/kg)
ARN22089 (12 mg/kg)

Figure S4. CDC42 inhibitors affect blood vessel arborization without inducing overt toxicity, related to Figure 4 and 5. (A) Micrographs of skin harvested from treated mice. Note the presence of observable vessels. (B) Skin of mice were treated with vehicle, vemurafenib, and ARN22089 at the indicated doses, optically cleared and imaged to generate Z-stacks. Flattened images from additional samples to those presented in Figure 4A are shown. Images were converted to grayscale and vessels traced with Angiotool. Scale bar is 100 μm. Each image (1.107 x 1.107 mm; 1024 x 1024 pixels) is from >50 flattened z-stacks (5 μm/stack). (C) Data from Figure 4B were replotted as averages of individual animal (unpaired two-tail *$p < 0.05$). Each dot corresponds to an individual animal with >4 images per animal. Data are represented as mean$\pm$SEM. (D) Scatterplot showing percent of live Cd31+ or Pdgfra+ skin and spleen cells (RhoJ-WT: vehicle vs 12 mg/kg, n=4 per group). Data are represented as mean$\pm$SD. (E) Skin from ARN22089 or vehicle treated mice was harvested for bulk RNAseq. Differentially expressed genes between ARN22089 and treated mice that play a role in endothelial cell or fibroblast biology (as identified by the PANTHER database) were plotted on a heatmap.

Figure S5

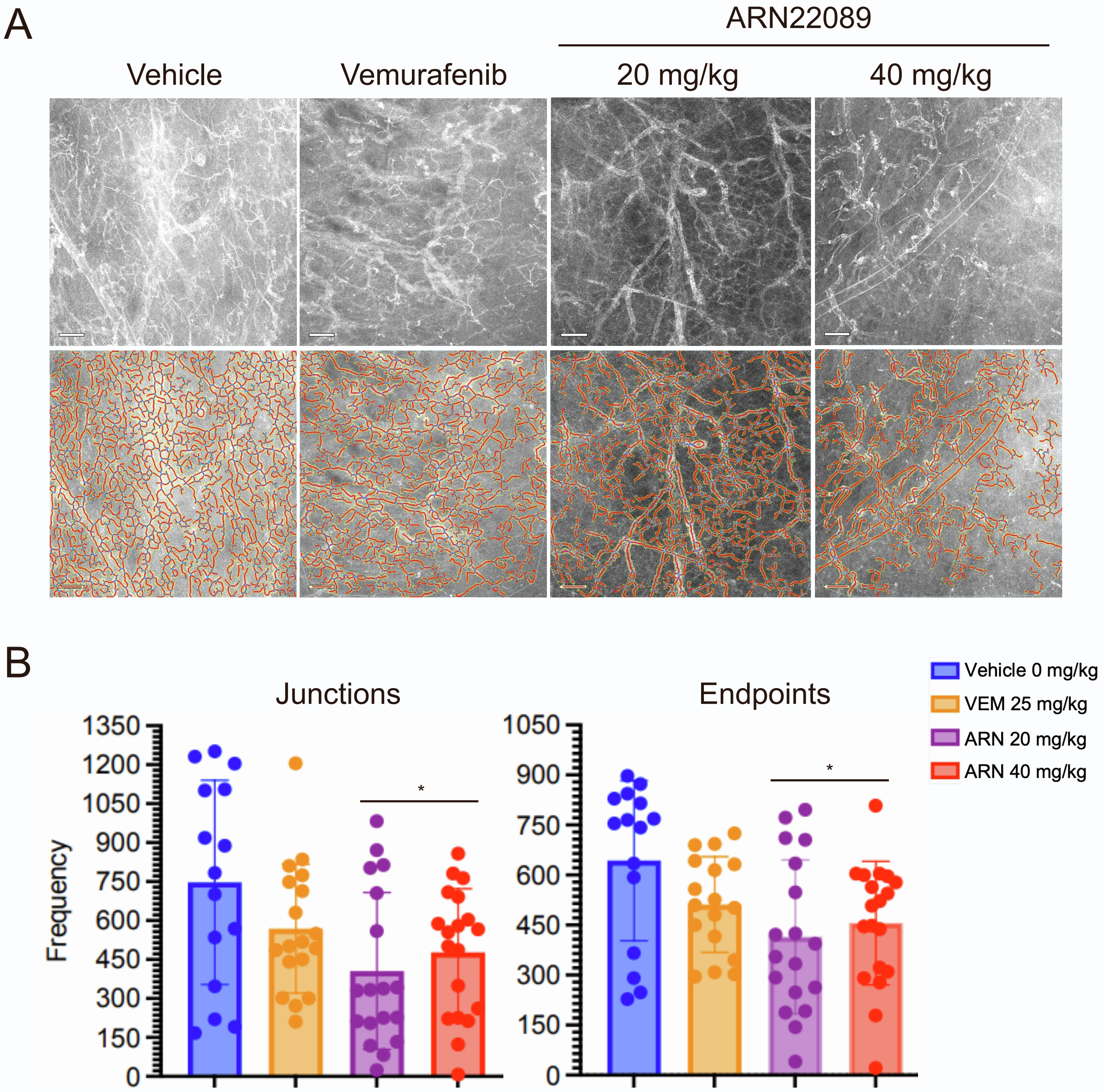
A
ARN22089
Vehicle
Vemurafenib
20 mg/kg
40 mg/kg
B
Junctions
Endpoints
Frequency
1350
1200
1050
900
750
600
450
300
150
0
1050
900
750
600
450
300
150
0
*
*
Vehicle 0 mg/kg
VEM 25 mg/kg
ARN 20 mg/kg
ARN 40 mg/kg

Figure S5. ARN222089 inhibits angiogenesis in skin adjacent to tumors in NSG mice, related to Figure 3. (A) Representative grayscale images of adjacent skin vessels from mice bearing tumors that were treated with 20 or 40 mg/kg twice a day of inhibitor for two weeks. Scale bar = 100 µm. Below shows 2D tracing with Angiotool. (B) Scatter plots show number of branches and vessel termini in mice treated with 20 and 40 mg/kg of ARN22089. Each image (1.107 x 1.107 mm; 1024 x 1024 pixels) has >50 z-stacks (5 µm/stack). Each dot corresponds to an individual image stack, *$p < 0.05$, >3 tumors per group were included. Data are represented as mean$\pm$SD.

Figure S6

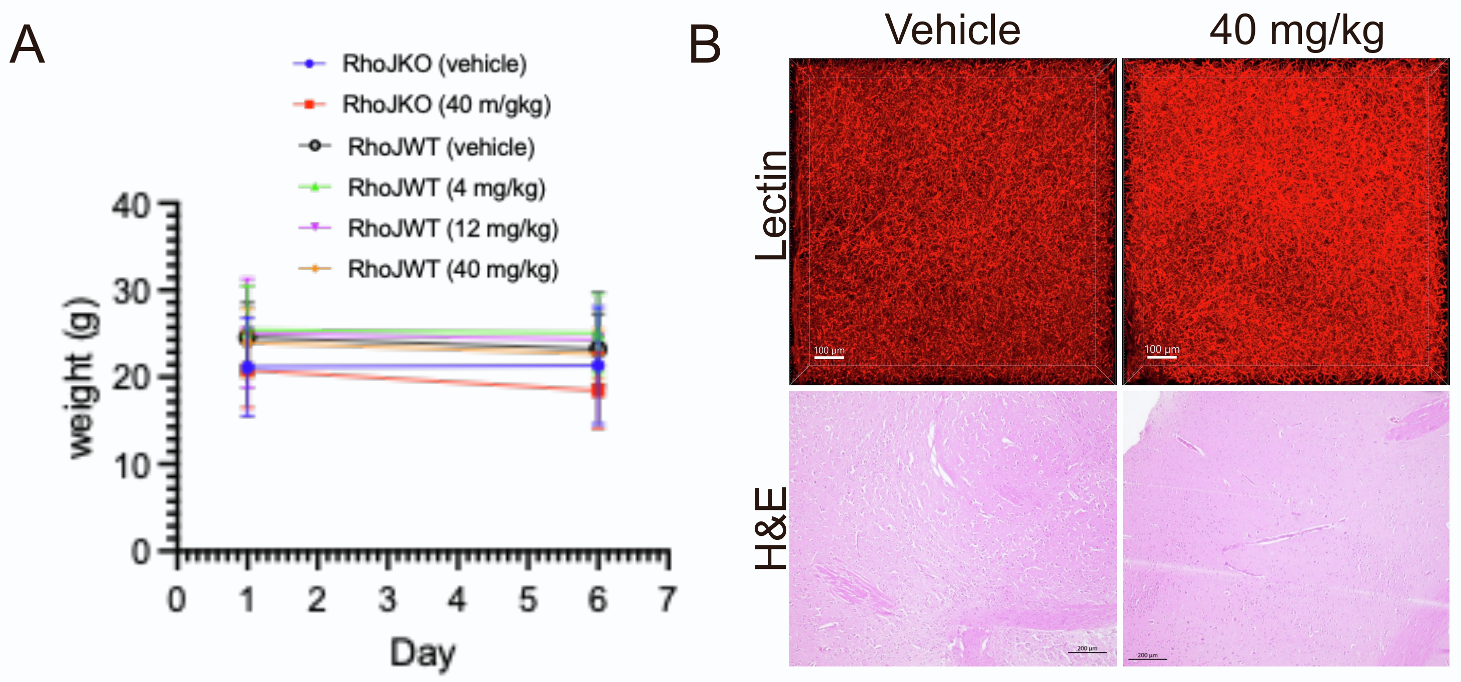

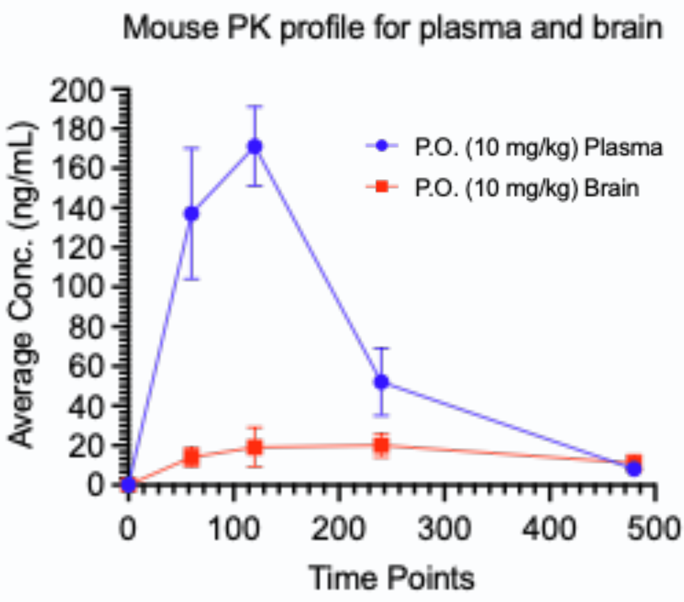

Time Points	P.O. (10mg/kg) Plasma		P.O. (10mg/kg) Brain	
min	Average conc. ng/mL	STD	Average Total Amount ng/Brain	STD
0	0	0	0	0
5	-	-	-	-
15	42	3	<LOQ*	-
30	110	20	<LOQ*	-
60	137	33	14	5
120	171	20	19	10
240	52	17	20	6
480	8	3	11	4

*Limit Of Quantification (LOQ = 5nM)

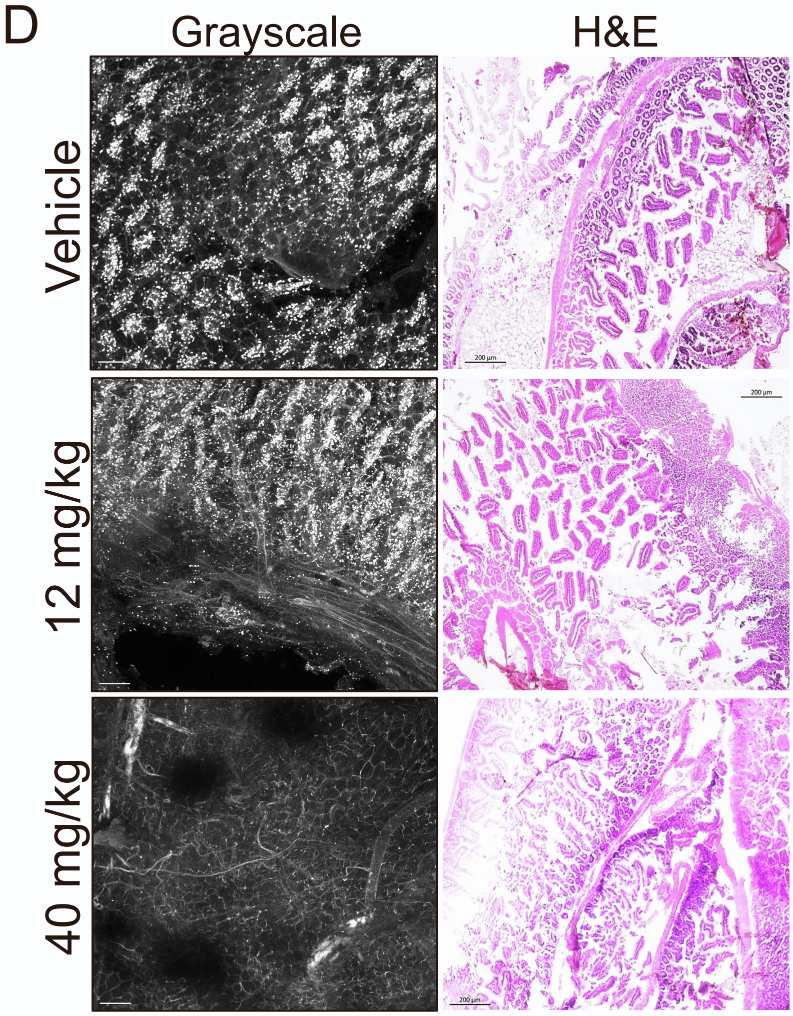

Figure S6. ARN22089 disrupts vessels in skin and colon but not the brain, related to Figure 2 and 4. (A) ARN22089 treatment does not affect mouse viability. Weights of mice treated with ARN22089 were measured daily; mean±SD. (B) Representative images of brain vessels in red labeled with lectin dyLight. Scale bar = 100 μm, from mice treated with the indicated doses of ARN22089 or vehicle. Representative H&E staining of brain tissue is shown. (C) PK profile of ARN22089 in the plasma and brain after oral administration of 10 mg/kg. The amount of compound detected in the brain was about 10 fold less than that in the plasma. (D) Representative images of cleared gastrointestinal tissues from mice treated with the indicated doses of ARN22089. Tissues were labeled with lectin dyLight, red fluorescence images were converted to grayscale using ImageJ. Decreased vessels were observed at the 12 and 40 mg/kg doses. Representative H&E staining of the intestinal tissues is shown, highlighting the decrease of villi in mice treated with 40 mg/kg of ARN22089.

Figure S7

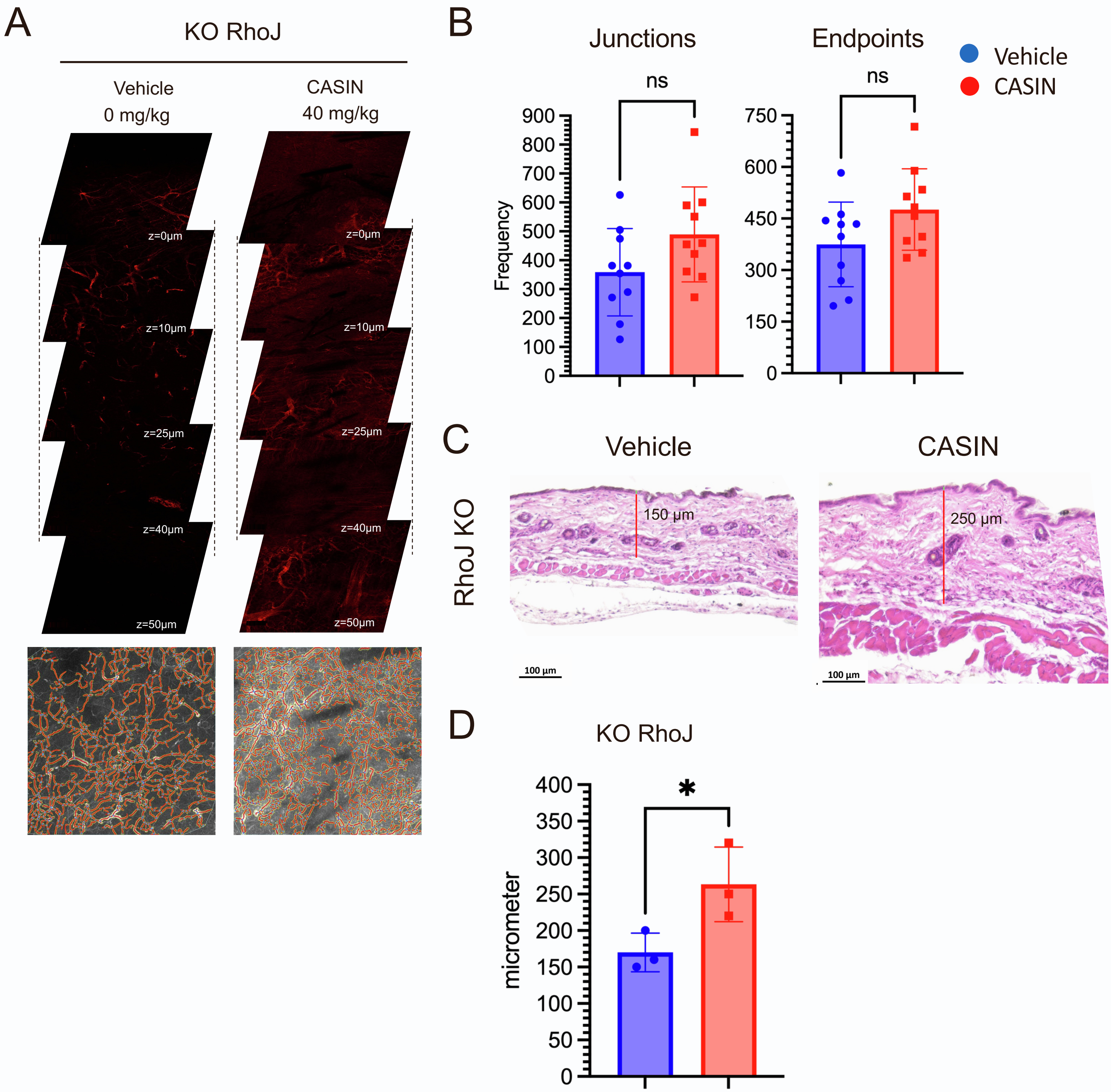

A
KO RhoJ
Vehicle
0 mg/kg
CASIN
40 mg/kg
z=0μm
z=10μm
z=25μm
z=40μm
z=50μm
B
Junctions
Endpoints
ns
ns
Frequency
900
800
700
600
500
400
300
200
100
0
750
600
450
300
150
0
Vehicle
CASIN
C
Vehicle
CASIN
RhoJ KO
150 μm
250 μm
100 μm
100 μm
D
KO RhoJ
*
micrometer
400
350
300
250
200
150
100
50
0

Figure S7. CASIN increase dermis thickness without affecting vessels through the inhibition of Cdc42 in a RhoJ knockout model, related to Figure 6. (A) Representative stack images of skin vessels from mice treated with vehicle or CASIN 40 mg/kg IP daily for one week. Bottom of each stack includes the 2D AngioTool tracing of pixels in grayscale. Scale bar is 100 µm. (B) Scatter plot with bar graphs show the frequency of branches, termini from flattened images using AngioTool. Unpaired t-test two tail was used to determine significance. Each dot corresponds to a Z stack images, n=3 animals per condition, $\geq$4 images per animal. (C) H and E staining of skin from wild type or knockout RhoJ mice treated with vehicle or CASIN at 40 mg/kg IP daily. Representative image is shown. (D) Quantification of the dermal thickness in drug and vehicle treated RhoJ knockout mice. 100 µm scale bar; *p-value < 0.05; n=3 per condition. Unpaired t- test two tail was used to determine significance. Data are represented as mean$\pm$SD.

Figure S8

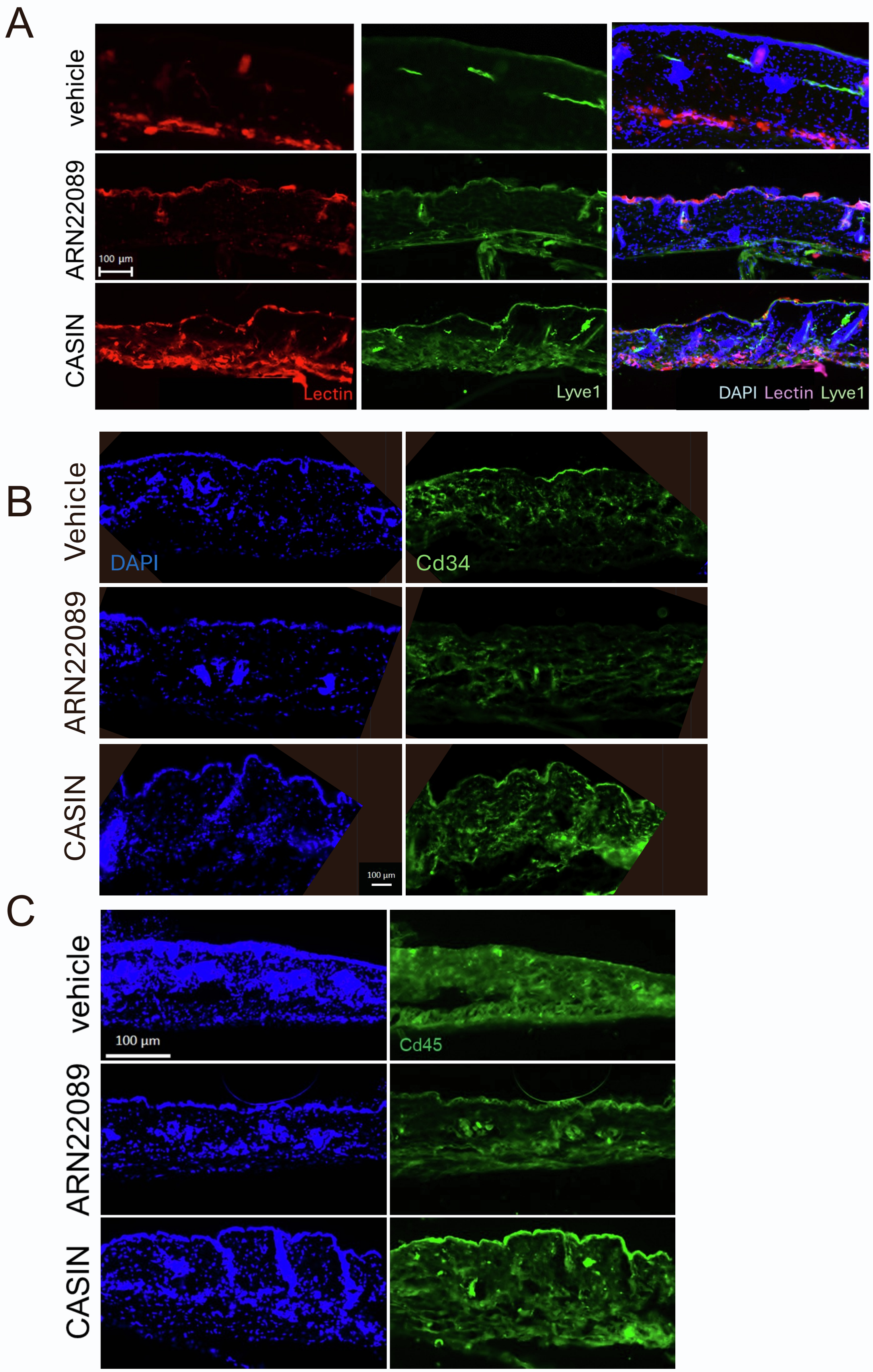
A
vehicle
ARN22089
CASIN
100 µm
Lectin
Lyve1
DAPI Lectin Lyve1
B
Vehicle
ARN22089
CASIN
DAPI
Cd34
100 µm
C
vehicle
ARN22089
CASIN
100 µm
Cd45

Figure S8. Expression of endothelial and immune cell marker genes are lower in ARN22089 treated skin as compared to vehicle or CASIN treated skin, related to Figure 5. Immunofluorescence of (A) Lyve1, (B) Cd34, and (C) Cd45 in skin of mice treated with vehicle, ARN22089 (40 mg/kg) or CASIN (40 mg/kg). Scale bar 100 μm. Lectin DyLight was used to visualize blood vessels in (A) and DAPI was used to counterstain the nuclei. Representative images from three biological replicates are shown.
